# Toward Efficient Hydrogen Production: Impact of Solid Solution of Tungsten on Nickel–Iron Hydroxide OER Catalysts

**DOI:** 10.1021/acscatal.5c07061

**Published:** 2026-02-06

**Authors:** Lamea Abbas, Lakhanlal, Sourav Bhowmick, Rawnaq Batheesh, Lior Elbaz, Maytal Caspary Toroker, Yoed Tsur

**Affiliations:** † The Wolfson Department of Chemical Engineering, 26747 Technion- Israel Institute of Technology, Haifa 3200003, Israel; ‡ Department of Materials Science and Engineering, 26747 Technion- Israel Institute of Technology, Haifa 3200003, Israel; § Department of Chemistry, Bar-Ilan Center for Nanotechnology and Advanced Materials, Bar-Ilan University, Ramat-Gan 5290002, Israel; ∥ Resnick Sustainability Center for Catalysis, Technion − Israel Institute of Technology, Haifa 3200003, Israel; ⊥ The Nancy and Stephen Grand Technion Energy Program, 26747 Technion- Israel Institute of Technology, Haifa 3200003, Israel

**Keywords:** doped nickel−iron catalysts, oxygen evolution reaction, electrocatalysis, water splitting, work function, density functional theory

## Abstract

Designing catalysts for the oxygen evolution reaction (OER) that are platinum group metal-free (PGM-free) is vital for making the production of hydrogen via water splitting more cost-effective. A trimetallic catalyst, NiFeW­(OH)_2_, was synthesized and studied using electrochemical methods, exhibiting higher catalytic performance than bare nickel–iron, manifested by faster reaction kinetics, evidenced by a lower Tafel slope and reduced effective resistance. This catalyst served as a parent compound for heat-treated catalysts in various conditions, such as air and inert atmosphere, to study the effect of the mixed oxide/hydroxide phase on electrochemical performance. X-ray Diffraction (XRD) revealed that tungsten addition expanded the crystal lattice by ∼30% in the c direction, which had a significant impact on the electronic environment, resulting in lowered binding energies, as revealed by X-ray photoemission spectroscopy (XPS). The most active composition was later studied in an anion exchange membrane water electrolyzer (AEM-WE) and showed high performance, reaching current densities of 2.12 A cm^–2^ at ∼2.0 V. Density functional theory (DFT) calculations assisted in identifying iron as the active site. Electrochemical impedance spectroscopy (EIS), analyzed by distribution function of relaxation times (DFRT, a.k.a. DRT), revealed the contribution of tungsten toward reduced charge transfer resistance. The best performances were found with compositions close to the solubility limit of tungsten in the system.

## Graphical abstract

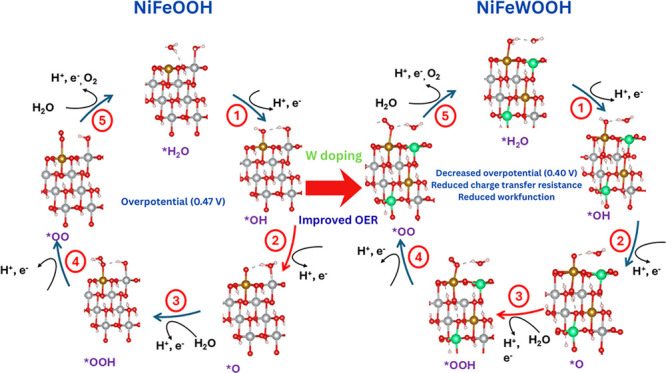

## 1 Introduction

The global shift toward renewable energy sources is accelerating, driven by growing environmental concerns and rising energy demands. Among the various green technologies, water splitting stands out as a crucial method for sustainable hydrogen production.^1–4^ This process involves two reactions taking place at the distinct electrodes. At the cathode, the hydrogen evolution reaction (HER) occurs, producing hydrogen by reducing the number of protons. Meanwhile, at the anode, the oxygen evolution reaction (OER) occurs, where water oxidation generates oxygen.^5,6^ The OER is a major bottleneck in this process due to its high overpotential and sluggish kinetics; thus, improving OER efficiency is essential for the viability of hydrogen production.^7^ Catalysts known to lower the activation energy barrier of a reaction lower the overpotential necessary to initiate OER, thereby increasing the process’s overall efficiency.^8,9^ The emphasis will be on transition metal-based catalysts in alkaline conditions, as most perform effectively primarily in this environment and tend to degrade under acidic conditions.^10^ Among these, nickel–iron hydroxide (NiFe) catalysts have demonstrated significant effectiveness for the OER due to the synergistic interaction between nickel and iron. Nickel is well-known for its faster redox reaction, while iron is known for faster charge transfer at the interface. So, incorporating Fe into nickel hydroxide increases the overall reaction kinetics due to improved charge transfer kinetics at the interfaces.^11–15^

While nickel–iron hydroxide catalysts are extensively researched for their OER effectiveness, their performance is often hindered by rapid degradation that can weaken the structure of the catalyst.^16^ Although NiFe is a step toward more affordable catalysts, significant improvements in its structural and functional properties are needed for it to be a practical choice for industrial use. To tackle these challenges, an effective strategy involves adding a third metal to enhance the performance of NiFe by improving its conductivity and modifying the structure of the binary catalyst.^14^ In this regard, tungsten is chosen for its distinctive electronic properties, its ionic size similarity to nickel, and its high valence states, making it a strong candidate to improve the catalytic performance of NiFe.^17^ Moreover, tungsten has already shown good performance in electrocatalysis, e.g., in nanodendritic alloys with Ir,^18^ in dual-doping of ruthenium oxide nanoparticles,^19^ in Ni–W alloy coating,^20,21^ and in high-entropy alloy nanoparticles.^22^ Tungsten incorporation in Ni-based electrocatalysts has been reported only in a few isolated cases, including bifunctional HER/OER catalysts based on W doping in Ni­(OH)_2_/NiOOH nanosheets,^23^ W-doped α-Ni­(OH)_2_,^24^ and W-doped Ni_12_P_5_ for the OER.^25^ In contrast, studies on W doping in Ni–Fe-based electrocatalysts are very limited. The specific role of W in NiFe systems and the cooperative interaction among NiFe and W, particularly its influence on the OER energetics, reaction pathways, and electronic structure, have not yet been systematically studied.

Electrochemical impedance spectroscopy (EIS) is a crucial characterization technique, widely used for its nondestructive property and ability to analyze reaction kinetics effectively. The EIS data are analyzed here using impedance spectroscopy genetic programming (ISGP).^26–29^ It transforms frequency dispersion into a distribution function of relaxation times (DFRT) to effectively infer physical properties. The DFRT involves a linear combination of known mathematical peaks, selected from a library, where ideally, each peak corresponds to a different electrochemical phenomenon. Upon identification of the different electrochemical phenomena within the system, their effective resistance and effective capacitance are extracted, considering the corresponding peak area and position.

Alongside experimental techniques to evaluate and analyze electrochemical performance, density functional theory (DFT) is extensively used to calculate various material properties of the catalyst^30–34^ and enables analysis of materials at the atomic level. With the help of DFT, the adsorption energy, overpotential, oxidation states, and work function can be calculated. Also, DFT explains the reasons for changes in overpotentials, which is an indicator of catalytic performance and helps to identify the active site of the reaction. It also provides insights into the electronic changes in the catalyst material caused by structural modifications or doping.

In this study, we have integrated and highlighted the significance of DFRT modeling and DFT calculations in understanding the effect of tungsten doping on the electrocatalytic activity and stability of the modified NiFe­(OH)_*x*_ catalysts. We synthesized samples with various stoichiometric ratios of Ni, Fe, and W and heat-treated them under air and N_2_ atmospheres, followed by their electrochemical characterizations. To gain further insights into the tungsten doping effect, DFRT and DFT analyses were conducted and compared with the experimental findings. Thus, this work helps us to understand the influence of tungsten doping on the electronic structure and electrochemical performance of the state-of-the-art doped NiFe­(OH)_*x*_ catalyst for water splitting under alkaline conditions. The solubility limit of tungsten in nickel–iron hydroxide is experimentally assessed, and it is also shown that a high concentration of tungsten is beneficial for the catalysis, although the tungsten itself is probably not the catalytic site.

## 2 Experimental Section

### 2.1 Reagents and Materials

Nickel­(II) chloride hexahydrate (NiCl_2_·6H_2_O, 99.3% trace metals basis; CAS: 7791–20–0) was purchased from Alfa Aesar. Iron­(II) chloride tetrahydrate (FeCl_2_·4H_2_O, 99.0% trace metals basis, CAS: 13478–10–9), ammonium metatungstate hydrate ((NH_4_)_6_H_2_W_12_O_40_·*x*H_2_O, 99.99% trace metals basis, CAS: 12333–11–8), potassium persulfate (K_2_S_2_O_8_, ACS reagent, ≥99.0%, CAS: 7727–21–1), ammonium hydroxide solution (NH_4_OH, ∼1 M NH_3_ in H_2_O, CAS: 1336–21–6), and ethanol (C_2_H_5_OH, 96%, CAS: 64–17–5) were purchased from Sigma-Aldrich. Isopropanol ((CH_3_)_2_CHOH, CAS: 67–63–0) was obtained from Gadot-Group. Nafion perfluorinated resin solution (5 wt % in lower aliphatic alcohols and water, contains 15–20% water, CAS: 31175–20–9), potassium hydroxide (KOH, ACS reagent, ≥85%, pellets, CAS: 1310–58–3), sodium hydroxide (NaOH, ACS reagent, ≥97.0%, pellets, CAS: 1310–73–2) were also obtained from Sigma-Aldrich. All the chemicals were used directly without any further treatment.

### 2.2 Electrocatalyst Synthesis

To obtain a total concentration of 0.1 M of precursor solution, nickel­(II) chloride hexahydrate, iron­(II) chloride tetrahydrate, and ammonium metatungstate hydrate were dissolved in 100 mL of distilled water with continuous stirring (350 rpm) at room temperature. At the same time, 1.25 mol of potassium persulfate, an oxidizing agent, was dissolved in 50 mL of distilled water and stirred for 15 min. After that, the potassium persulfate solution was added dropwise to the metal salt solution, and the mixture was stirred for 10 min. Then, 5 mL of an ammonium hydroxide solution was added to the solution gradually to create an alkaline environment necessary for the precipitation of the metal ions. Thereafter, the solution was kept under continuous stirring at room temperature. After complete dissolution, the solution was kept undisturbed for 24 h at room temperature for sedimentation of the formed hydroxide molecules. The sedimented particles were then washed 3 times with double-distilled water and ethanol (96%) by centrifugation to remove unreacted species. Finally, the particles were dried at 60 °C overnight and ground to form a fine powder of the catalysts.

The NiFeW served as the parent compound for synthesizing the heat-treated catalysts. It was placed in an alumina boat in a programmable tube furnace for consistent heating. To synthesize the NiFeW-Air-5 °C catalyst, the sample was heated to 400 °C for 2 h in air with a heating/cooling rate of 5 °C min^–1^. After it was cooled, the sample was ground. Other combinations of the atmosphere and heating/cooling rates were also tried. In particular, the NiFeW-N_2_-2 °C catalyst was prepared using the above procedure, but in a nitrogen atmosphere with a heating/cooling rate of 2 °C min^–1^.

### 2.3 Catalyst Ink Preparation

The catalyst ink was prepared by mixing 5 mg of the as-synthesized catalyst with 750 μL of ultrapure (>18.2 MΩ cm) deionized water, 250 μL of isopropanol, and 45 μL of Nafion. This mixture was sonicated for 3 h at room temperature to generate a homogeneous solution. Subsequently, 8 μL of the ink was drop-cast onto the active area of a glassy carbon electrode (GCE) with a 5 mm inner diameter. The fabricated GCE was then dried at 30 °C overnight.

### 2.4 Electrochemical Measurements

The electrochemical measurements were conducted utilizing a rotating disc electrode (RDE) system (RRDE-3A, ALS Co., LTD) with a three-electrode system rotating at 1600 rpm. The fabricated GCE was used as the working electrode, a glassy carbon rod as the counter electrode, and mercury/mercuric oxide (Hg/HgO) filled with 1 M NaOH was used as the reference electrode. The electrolyte system was 0.1 M KOH, creating an alkaline environment. The RDE was assembled with a VSP potentiostat (BioLogic) to determine the electrochemical performance of the catalyst.

Prior to electrocatalytic testing, each catalyst was activated by conducting 100 cycles of cyclic voltammetry (CV) at a scan rate of 10 mV s^–1^ at a potential range of 0.2–0.8 V vs Hg/HgO. Using chronoamperometry (CA), the stability of the catalysts was assessed over 20 h at an overpotential of 300 mV (1.53 V vs RHE). The polarization curve for the catalyst was obtained using linear sweep voltammetry (LSV) at a scan rate of 5 mV s^–1^ at a potential range of 0.2–0.8 V vs Hg/HgO, both before and after the stability test. EIS measurements were conducted both before and following the stability test at a frequency range of 0.1–10^5^ Hz at an overpotential of 300 mV. All the obtained potentials are calculated relative to the reversible hydrogen electrode (RHE) using the Nernst equation with an *iR* correction: *E*_RHE_ = *E*_measured_ + 0.14 + 0.059 × pH – *iR*, where *R* is the solution series resistance, as found from the high-frequency edge of the EIS measurements. This can be found with high accuracy by ISGP.^35^

### 2.5 Impedance Spectroscopy Analysis by Genetic Programming

ISGP was used to analyze the EIS data. The DFRTs obtained from ISGP for all catalysts were subsequently compared and discussed in Section 3. To determine the most suitable DFRT, two identical sets of EIS impedance data, differing only in noise, were inserted as input to the ISGP. Then, the Kramers–Kronig (K–K) transformation was used to evaluate the validity of the data. From the DFRT plot, we determine the effective resistance and effective capacitance of each electrochemical phenomenon for the respective catalysts. This includes effective double-layer capacitance and charge transfer. The effective resistance is calculated by multiplying the area under the corresponding peak by the normalization factor. The effective capacitance can be calculated by dividing the central relaxation time (τ) of the process by the effective resistance.

### 2.6 Electrolyzer Measurement

The cathode was composed of 0.5 mg cm^–2^ of commercial Pt–Ru/C (Pt 20%, Ru 10% on graphitized carbon, SIGMA-Aldrich), and the anode of NiFeW was composed of 2 mg cm^–2^ on nickel foam. The membrane used was a PiperION Anion Exchange Membrane, 40 μm (Versogen, PiperION-A-20-HCO3). The membrane was activated by leaving it open to air for an hour, then soaking it in 0.5 M NaOH for 2 h with a solution exchange after the first hour. The membrane electrode assembly (MEA) was constructed using gaskets on both sides and assembled without hot pressing, with the cell tightened to 7 N m. The cell was then tested in a Scribner 600 Electrolyzer Test System. It was operated at 70 °C, with 1 M KOH flowing on both the anodic and the cathodic sides at a rate of 40 mL min^–1^. For the break-in, the cell voltage was cycled once between 1.3 and 2.0 V and held at 0.1 A for 10 min. The polarization curve was then obtained, holding for 3 min at each current and recording the voltage every 50 s.

### 2.7 DFT Study

Spin-polarized DFT calculations were performed using VASP (version 5.4.4).^36,37^ A plane wave energy cutoff of 600 eV is used. In the calculation Hubbard U-J parameter of 5.5 and 3.3 eV is used for Ni and Fe, respectively.^38^ To sample the Brillouin zone, a 2 × 2 × 1 gamma-centered *k*-point grid was used. The energy cutoff and *k*-point grid were converged to within <1 meV. Projected-augmented wave (PAW) potentials were used to replace the core electrons of Ni (1s, 2s, 2p, 3s, 3p), Fe (1s, 2s, 2p, 3s, 3p), W (1s, 2s, 2p, 3s, 3p, 3d, 4s, 4p, 4d, 4f, 5s, 5p), and O (1s).^39^

For this study, the slab model is made by cleaving the β-NiOOH surface at (0 $\overset{-}{1}$ 5) plane, considering that this plane is believed to be chemically active and commonly reported in literature.^23,38,40–42^ Also, using (0 $\overset{-}{1}$ 5) surface makes our result comparable with results reported in existing literature. After making the slab model for NiOOH, Ni sites were substituted with Fe and W to mimic different catalysts. The slab model had 12 Ni atoms. In the slab, each layer contains two metal atoms. To mimic Ni_0.9_Fe_0.1_OOH, one Ni site at top of the slab is replaced with Fe. In the experiment, the best-performing sample was Ni_0.58_Fe_0.16_W_0.26_OOH, and to closely mimic that, we replaced two Ni atoms with Fe and three Ni atoms with W. Here, the top layer becomes most important as catalysis is going to take place there. For the composition of Ni_0.59_Fe_0.16_W_0.25_OOH, we considered four different cases. In the first case (NiFeWOOH-**Fe**Ni), the top layer contains Fe and Ni sites, and catalysis occurs on the Fe site. The other three cases are NiFeWOOH-**W**Fe, NiFeWOOH-W**Fe**, and NiFeWOOH-Fe**Ni**. In the code for the material, the letters after "-" show the sites of the top layer, and the letter marked in bold denotes the active sites. The nomenclature of the materials is given in Table 1.

**Table 1:** Details of the Material’s Composition Considered for Computation

**experimental material**	**computational material**	**code**	**top-layer atoms**	**active site**
Ni_0.9_Fe_0.1_OOH	Ni_0.92_Fe_0.08_OOH	NiFeOOH	Ni, Fe	Fe
Ni_0.58_Fe_0.16_W_0.26_OOH	Ni_0.59_Fe_0.16_W_0.25_OOH	NiFeWOOH-**Fe**Ni	Ni, Fe	Fe
	Ni_0.59_Fe_0.16_W_0.25_OOH	NiFeWOOH-W**Fe**	W, Fe	Fe
	Ni_0.59_Fe_0.16_W_0.25_OOH	NiFeWOOH-**W**Fe	W, Fe	W
	Ni_0.59_Fe_0.16_W_0.25_OOH	NiFeWOOH-Fe**Ni**	Fe, Ni	Ni

For the OER, the overpotential is calculated for all the above-mentioned materials in Table 1. In the slab model, at the top, there are two exposed metal atoms. Depending on the materials, the exposed atoms could be Ni, Fe, or W. Also, the OER occurs only on a single metal site, and the other site always has an adsorbed water molecule, as done in previous studies for OER on NiOOH. A similar reaction mechanism has been used in the literature to study OER.^43^ The other adsorbed spectator water molecule introduces a solvent effect at the micro level, and this model has already been used.^44,45^

To further study the charge distribution after doping, Bader charge analysis is also used along with the charge density difference analysis. The density of states (DOS) and the d-band center are also calculated in this study. Work functions were also calculated to study the charge transfer aspects of these catalysts.

The optimized structure files, along with the other relevant data such as Bader charge, magnetization, etc., for all DFT calculations, are provided as a zipped folder in the Supporting Information.

## 3 Results and Discussion

### 3.1 Physicochemical Characterizations of the Catalysts

In a preliminary study, we utilized thermogravimetric analysis (TGA) to identify the optimal temperature for the thermal treatment of our catalysts. TGA enabled the precise determination of the temperatures at which significant weight losses occurred, indicating the removal of water molecules and the decomposition points of the catalysts. Based on our findings (Figure 1a), we selected temperatures of 270 and 400 °C for conducting the heat treatment in air with heating/cooling rates of 2 °C/min or 5 °C min^–1^ for each temperature. The choice of 270 °C was due to the significant weight loss observed, primarily from the desorption of water. The temperature of 400 °C was chosen because the weight loss was nearly complete, indicating the decomposition of the hydroxide phase to the oxide phase via the release of water molecules.^46^ For the N_2_ atmosphere, we selected a temperature of 400 °C with a heating/cooling rate of 2 °C min^–1^. This decision was based on previous research within our group, demonstrating optimum catalyst performance under similar conditions.^46^ Finally, based on the LSV curves of all the as-synthesized catalysts, shown in the Supporting Information (Figures S1–S3), we selected the catalyst that underwent heat treatment. This catalyst demonstrated the most effective electrochemical performance. See Figure S4 in the Supporting Information for DTA, which confirms that the weight loss results from an endothermic reaction.

**Figure 1:**
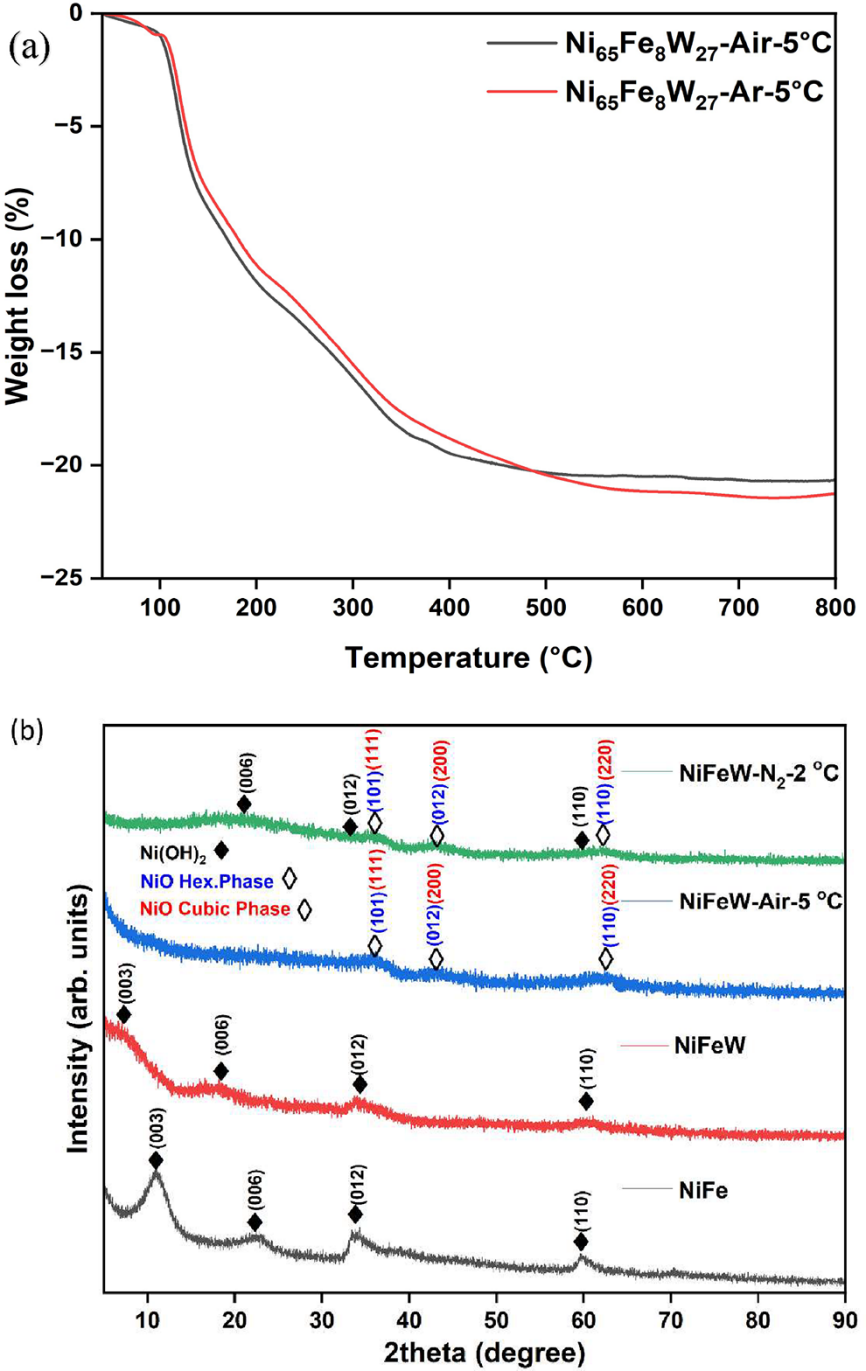
(a) TGA curves of Ni_65_Fe_8_W_27_ in air and argon (Ar) atmospheres. (b) XRD patterns of the catalysts: NiFe, NiFeW, NiFeW-Air-5 °C, and NiFeW-N_2_-2 °C. For the NiO phase, possible indices for both the cubic and hexagonal phases are presented; see the text.

The crystal phases of the catalysts were analyzed with an X-ray diffraction (XRD) technique. Figure 1b shows the XRD patterns of NiFe, NiFeW, NiFeW-Air-5 °C, and NiFeW-N_2_-2 °C. The obtained XRD peaks of NiFe and NiFeW were matched with the hexagonal phase of Ni­(OH)_2_ (JCPDS file no. 00–038–0715). This confirms the successful metal doping into the Ni­(OH)_2_ lattice. The first two peaks, (003) and (006), in the NiFeW sample show a significant shift compared with those in the NiFe specimen. This shift suggests an increased *c* parameter, indicating the incorporation of interlayer species, such as water molecules or interstitial hydroxide ions, within the interlayers of the structure.^47^ In NiFeW-Air-5 °C, the peaks correspond to the oxide phase of Ni (NiO, either the hexagonal phase (JCPDS file no. 00–044–1159), or the cubic phase (JCPDS file no. 00–047–1049). It is impossible to determine the exact phase (cubic or hexagonal) of NiO from the three main peaks. Cubic (111) can also be hexagonal (101), cubic (200) can be hexagonal (012), and cubic (220) can be hexagonal (110). However, in the NiFeW-N_2_-2 °C case, peaks corresponding to the hexagonal phase of Ni­(OH)_2_ are observed, along with additional peaks of the NiO phase due to the formation of oxide/hydroxide mixed phases. The XRD spectra of several other synthesized materials are presented in Figure S5 of the Supporting Information. In some catalysts, impurity peaks were detected, allowing us to determine the solubility limit of tungsten in the system, which is approximately 27%.

Scanning electron microscopy (SEM) was employed to examine the surface morphologies of the synthesized catalysts. Figure 2 illustrates the SEM images of NiFe, NiFeW, NiFeW-Air-5 °C, and NiFeW-N_2_-2 °C. The four as-synthesized catalysts (Figure 2a–d) are nanoparticles with an average size of ∼5 nm. However, while this may suggest that doping and heat treatment do not considerably affect the nanostructure of the catalysts, indicating retention of the surface morphology, the BET results (Table 1) do indicate a change in the surface area of the catalysts.

**Figure 2:**
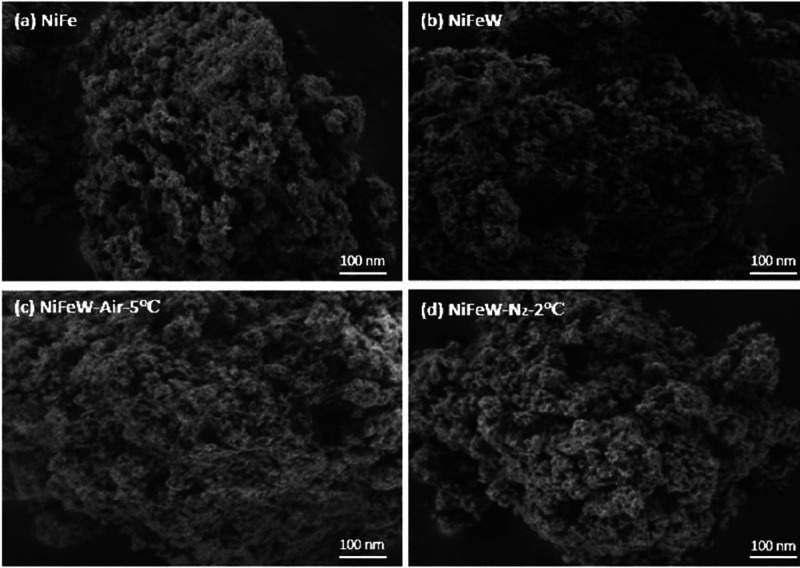
High-resolution scanning electron microscopy images: (a) NiFe; (b) NiFeW; (c) NiFeW-Air-5 °C; and (d) NiFeW-N_2_-2 °C.

The surface area and total pore volume of materials were measured using BET. From Table 2, the undoped NiFe catalyst shows the highest surface area. The surface area decreases with tungsten doping and subsequent heat-treatments (HT). See Figures S6 and S7 in the Supporting Information for nitrogen adsorption–desorption curves and BJH desorption cumulative pore volume plots of the catalysts, respectively. Tungsten doping in nickel–iron hydroxides alters the electronic properties of the catalyst. This doping can change the oxidation states and the electronic density near the active sites, potentially lowering the energy barrier for water oxidation reactions. This effect could enhance the intrinsic activity per active site, making the catalyst more efficient, even with a lower total surface area.

**Table 2:** N_2_ Physisorption Results at 77 K for All Catalysts

**catalyst**	**BET surface** **area [m** ^**2**^ **g** ^**–1**^ **]**	**total pore** **volume [cm** ^**3**^ **g** ^**–1**^ **]**	**average pore diameter [Å]**
NiFe	274	1.13	166
NiFeW	180	0.67	150
NiFeW-Air-5 °C	148	0.45	121
NiFeW-N_2_-2 °C	129	0.54	167

X-ray fluorescence (XRF) measurements were conducted to quantify the elemental composition of the catalysts (Table 3). The observed discrepancies between the expected and actual metal ratios in the catalysts can primarily be attributed to the challenges associated with controlling the chemical reactions in solution, particularly when incorporating tungsten as the third metal. Differences in the solubilities of the various cations in the solution complicate the precise control over reactant concentrations and environmental conditions. Interestingly, this happened only in W-containing systems. Additionally, the results indicate that heat treatment does not alter the atomic% of the respective elements.

**Table 3:** XRF of the Electrocatalysts with Atomic%

**sl. no.**	**compound**	**Ni (At%)**	**Fe (At%)**	**W (At%)**
1	Ni_90_Fe_10_	90	10	0
2	Ni_65_Fe_8_W_27_	58	16	26
3	NiFeW-Air-5 °C	58	16	26
4	NiFeW-N_2_-2 °C	58	16	26
5	Ni_80_Fe_9_W_11_	71	14	15
6	Ni_53_Fe_7_W_40_	40	11	49

To further explore the impact of tungsten solid solution on NiFe­(OH)_2_/NiFeOOH, X-ray photoelectron spectroscopy (XPS) was utilized to gain insights into the valence states of each element within the catalyst. Figure 3 displays the XPS results of NiFe, NiFeW, NiFeW-Air-5 °C, and NiFeW-N_2_-2 °C. Figure 3a shows the core-level spectra of Ni 2p with binding energies of Ni 2p_3/2_ and Ni 2p_1/2_ for NiFe and NiFeW-Air-5 °C, centered at 855.97 and 873.68 eV, respectively.^17,48^ Shifts in the binding energies are observed for NiFeW and NiFeW-N_2_-2 °C, with binding energies of 856.06 and 873.59 eV for NiFeW and 855.88 and 873.68 eV for NiFeW-N_2_-2 °C. The Ni 2p_3/2_ spectrum is deconvoluted into two peaks: the green peak indicating Ni^2+^ and the purple peak indicating Ni^3+^. Additionally, there is a corresponding satellite peak with two smaller peaks (yellow and blue). The Ni 2p_1/2_ peak is also deconvoluted into two peaks: the brown peak indicates Ni^2+^, and the dark blue peak indicates Ni^3+^, with a satellite peak at 879.29 eV (orange).^17,48^ The presence of both Ni^2+^ and Ni^3+^ across all catalysts indicates a mixed oxidation environment for Ni. The pink peak in NiFeW-Air-5 °C, at binding energies of 851.85 eV, refers to metallic nickel.^49^

**Figure 3:**
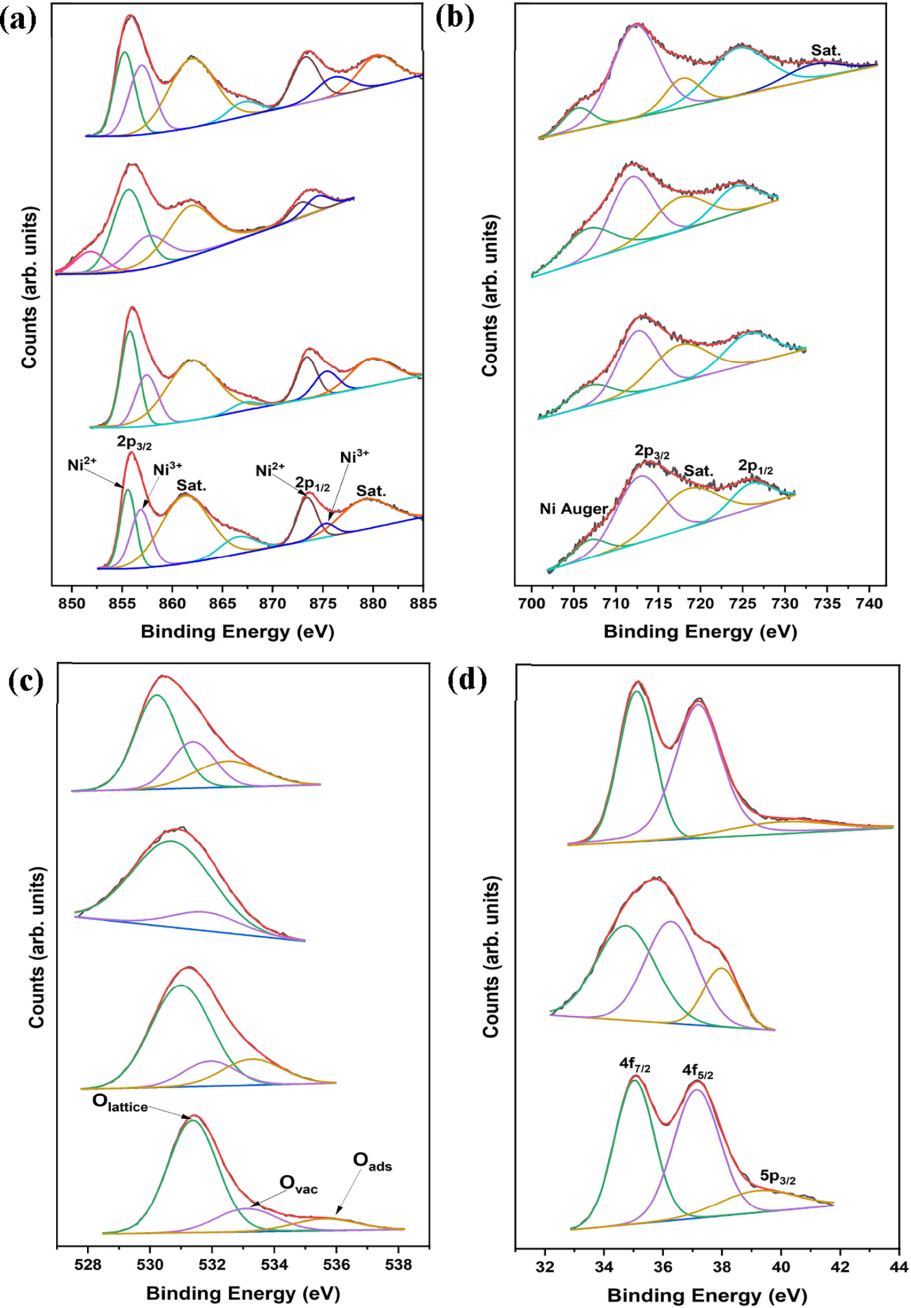
Deconvoluted high-resolution X-ray photoelectron spectra of, from the bottom upward: NiFe, NiFeW, NiFeW-Air-5 °C, and NiFeW-N_2_-2 °C: (a) Ni 2p; (b) Fe 2p; (c) O 1s; (d) W 4f.

In Figure 3b, the core-level Fe 2p spectra with binding energies for Fe 2p_3/2_ and Fe 2p_1/2_ in the NiFe catalyst are recorded at 713.50 and 726.12 eV, respectively. Tungsten-doped catalysts exhibit lower binding energies: 713.17 and 725.91 eV for NiFeW, 711.76 and 724.59 eV for NiFeW-Air-5 °C, and 712.63 and 724.80 eV for NiFeW-N_2_-2 °C. In all the catalysts, the green peak is a Ni Auger peak.^46^ The Fe 2p_3/2_ (purple) and Fe 2p_1/2_ (blue) peaks indicate Fe^3+^, with a corresponding satellite peak (yellow).^46,50^ An additional satellite peak (dark blue) is observed in NiFeW-N_2_-2 °C, which is not visible in other samples due to lower binding energy measurements.

Figure 3c shows the core-level O 1s spectra with binding energy in the NiFe catalyst at 531.38 eV (green), corresponding to the lattice oxygen (O_lattice_).^50–52^ In the NiFeW catalyst, the binding energy for O_lattice_ decreases slightly to 531.01 eV, continuing to 530.65 eV in NiFeW-Air-5 °C and 530.29 eV in NiFeW-N_2_-2 °C. The purple peak, indicating oxygen vacancies (O_vac_),^17^ appears at 533.13 eV for NiFe, 531.96 eV for NiFeW, 531.79 eV for NiFeW-Air-5 °C, and 531.52 eV for NiFeW-N_2_-2 °C. A significant increase in oxygen vacancies is observed in NiFeW-N_2_-2 °C due to heat treatment in an inert atmosphere with a scarcity of oxygen. The yellow peak represents adsorbed water (O_ads_),^50,51^ appearing at 535.66 eV for NiFe, 533.32 eV for NiFeW, and 532.41 eV for NiFeW-N_2_-2 °C. The presence of tungsten increases the hydrophilicity of the NiFeW catalyst, as evidenced by the larger peak area for adsorbed water.

In Figure 3d, the core-level 4f spectra of tungsten with binding energies of W 4f_7/2_ and W 4f_5/2_ for NiFeW are located at 35.05 eV (green peak) and 37.16 eV (purple peak), respectively. In the NiFeW-N_2_-2 °C sample, these values shift slightly to 35.11 and 37.22 eV. These slight changes suggest that the valence state of W remains consistent across these catalysts, +6.^48^ In the NiFeW-Air-5 °C sample, these values shift to 34.69 and 36.26 eV. Despite these shifts in binding energy, the tungsten maintains a +6 oxidation state, suggesting that while the chemical environment of tungsten may have been affected by the air exposure, its valence state remains unchanged.^53^ The third peak, associated with 5p_3/2_, indicates also the presence of W^6+^, which was observed at 39.36 eV for NiFe, 37.94 eV for NiFeW-Air-5 °C, and 40.20 eV for NiFeW-N_2_-2 °C.^50^

The XPS analysis reveals that the incorporation of tungsten and subsequent heat treatments affect the iron and oxygen in the catalysts without significantly altering the chemical state of nickel. The consistent decrease in iron and oxygen binding energies suggests a reduction in their oxidation states or a shift toward a more electron-rich environment, potentially enhancing catalytic activity.^17^ Tungsten maintains its oxidation state at +6 across all conditions with a red shift in binding energy observed during air exposure. These findings highlight the sensitivity of iron and oxygen to structural modifications and environmental conditions, underscoring their roles in the catalytic performance and stability of these materials. Furthermore, from the XRD results, we can deduce that the increase in the c parameter in the lattice for the tungsten-doped catalysts corresponds to a decrease in the binding energy. This suggests that changes in the crystal lattice structure, specifically the apparent sole expansion in the *c* direction, influence the electronic environment, leading to decreased binding energies.^54^ This interpretation bridges the structural insights provided by XRD with the electronic and chemical state information provided by XPS.

### 3.2 Electrocatalytic Activity

To evaluate the OER activities of the electrocatalysts, a three-electrode cell was utilized, consisting of a catalyst-modified GCE as the working electrode, a GCE as the counter electrode, and a Hg/HgO filler filled with 1 M NaOH as the reference electrode, all within a 0.1 M KOH electrolyte. The electrolyte was continuously purged with nitrogen to remove dissolved oxygen from the water. Initially, the catalysts were activated through 100 CV cycles ranging from 0.2 to 0.8 V vs Hg/HgO at a scan rate of 10 mV/s. During these activation cycles, a segment of the hydroxide phase was transformed into oxyhydroxide.^7,14,55^

Figure 4 shows the LSV curves with *iR*-correction before and after the stability test of the HT catalysts, in comparison with the parent catalysts. The oxidation peak observed in all the measurements (∼1.45 V vs RHE) signifies the oxidation of Ni^2+^ to Ni^3+^.^13,14,46^ Figure S8 shows the comparative LSV curves for all of the synthesized catalysts, which indicates that the most effective catalyst containing tungsten is Ni_65_Fe_8_W_27,_ which exhibits superior electrochemical performance. To investigate the influence of HT on the electrochemical performance of the catalysts, the most efficient catalyst (Ni_65_Fe_8_W_27_) was selected as the parent compound for HT.

**Figure 4:**
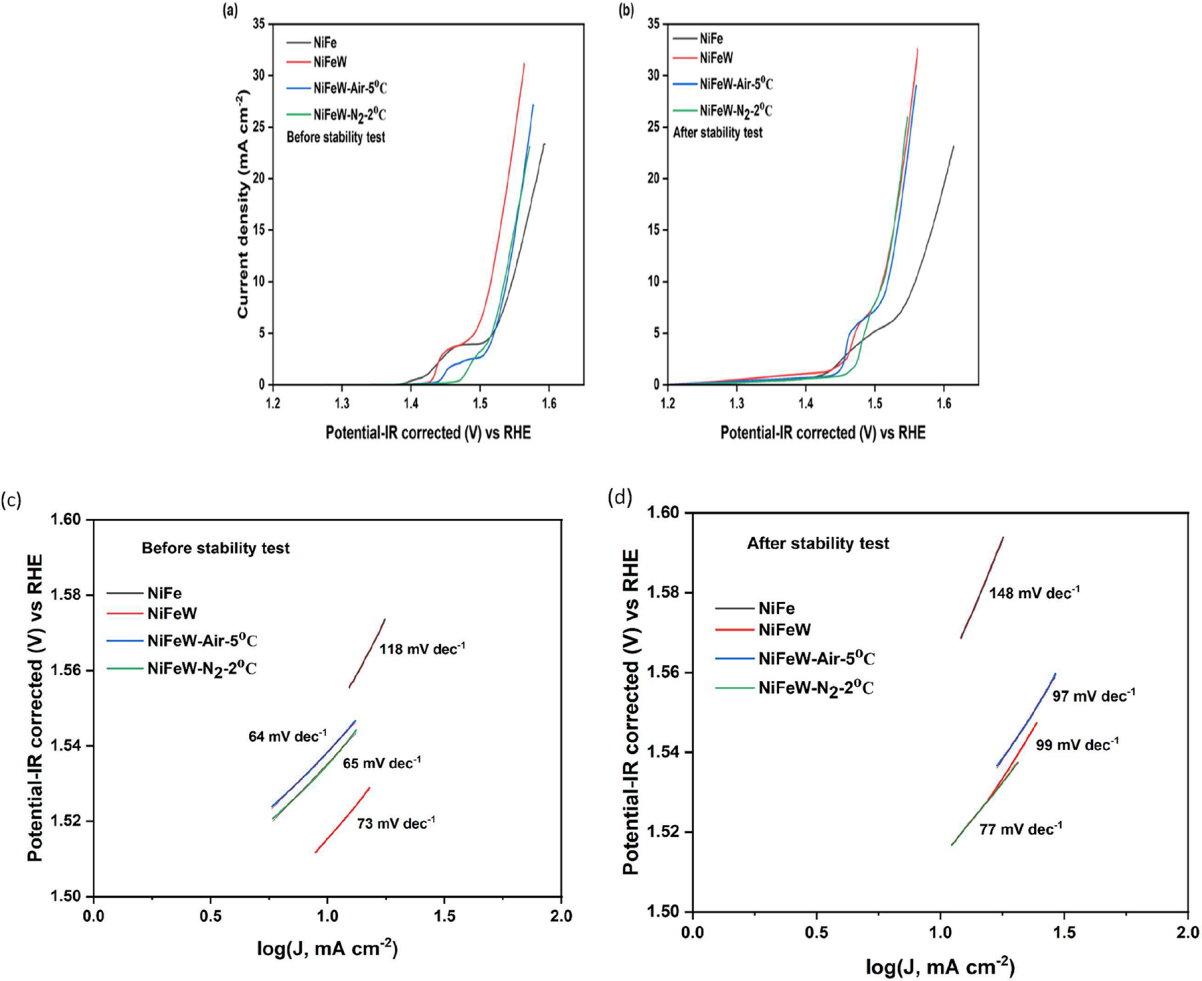
Polarization curves of NiFe, NiFeW, NiFeW-Air-5 °C, and NiFeW-N_2_-2 °C: (a) before and (b) after the stability test. Tafel slopes of all the catalysts: (c) before and (d) after the stability test.

It is observed for both before and after stability tests that the NiFeW catalyst shows better performance than the bare NiFe with overpotentials of 290 and 280 mV compared to 320 and 330 mV at 10 mA/cm^2^. The tungsten addition to NiFe increases the redox cycle kinetics of Ni, thereby improving the OER process. The tungsten addition to the NiFe catalyst resulted in the formation of a Ni–O–W bond.^48^ The W^6+^ has vacant d-orbitals, which act as electron acceptors. The electron from the Ni d-orbital moves to the W vacant d-orbitals via the oxygen 2p orbital.^48,56^ As a result, the Ni^2+^ partially oxidizes to the Ni^(2+δ)+^ state, and further oxidation to Ni^3/4+^ during the OER becomes much easier. Thus, the rate of the redox cycle increases due to W^6+^ addition, which, in turn, facilitates the OER mechanism. In addition to facilitating the OER mechanism, the W^6+^ is also known to reduce the water dissociation energy and improve the adsorption energy for the intermediates, which facilitates the formation of O_2_ molecules.^23,48^

In Figure 4a, the polarization curves (before the stability test) of NiFeW after heat treatments show a shift in the oxidation peak. This indicates an increase in the oxidation potential for Ni^2+^ to Ni^3+^. Furthermore, the current density decline suggests a reduction in reaction kinetics due to the formation of the oxide/hydroxide mixed phase. After the stability tests, it is observed that the polarization curve (Figure 4b) of bare NiFe shows performance deterioration, which may be due to possible Fe leaching, compromising its stability.^16^ However, after comparing the heat-treated catalysts after the stability test, the oxidation peak has almost the same potential as that of NiFeW.

Figure 4c shows the calculated Tafel slope using the Tafel equation: η = *b* × log­(*i*) + *a*, where η is the overpotential (V), *b* is the Tafel slope (V/decade), *i* is the current density (mA cm^–2^), and *a* is a constant related to the exchange current density. Usually, an effective electrocatalyst demonstrates a low Tafel slope value owing to a faster charge transfer coefficient.^57^ Doping tungsten significantly lowered the Tafel slope value compared with the NiFe catalyst, indicating faster kinetics. It is seen that the Tafel slope of NiFeW further reduced upon heat treatment, indicating much faster reaction kinetics at the electrolyte interface for the mixed oxide/hydroxide phase. In Figure 4d, the Tafel slope of all the catalysts is shown after the stability test. It is seen that the Tafel slope value increased, indicating a lowering of the reaction kinetics.

The overpotentials and Tafel slopes of each catalyst are shown in Table 4. The performance enhancement after the stability test may be due to the activation of electroactive sites of the oxide/hydroxide mixed-phase catalyst during the long stability run.

**Table 4:** Overpotential and Calculated Tafel Slopes of Each Catalyst

**catalyst**	**overpotential [mV] at 10** mA cm^**–2**^ **before**	**Tafel slope [mV/dec] before**	**overpotential [mV] at 10** mA cm^**–2**^ **after**	**Tafel slope [mV/dec] after**
NiFe	320	118	330	148
NiFeW	290	73	280	99
NiFeW-Air-5 °C	310	64	290	97
NiFeW-N_2_-2 °C	310	65	280	77

However, the total current density of the catalysts subjected to heat treatment does not increase compared to that of the catalyst before the heat treatment. This may originate from the fact that there is an oxide phase besides the hydroxide. The oxide phase, although stable compared to that of the hydroxide, does not react actively within a short time. We can see from the polarization curves after the stability test that the current density has increased.

Figure 5 shows the stability test (CA curves) spanning 20 h at an overpotential of 300 mV. The current density of all the catalysts rises over time, indicating an improvement in catalytic activity. The wavy curve for the NiFe catalyst is due to attachment of formed O_2_ on the catalyst surface. In W-doped NiFe, the wave curves decrease, indicating enhanced surface hydrophilicity upon doping and leading to easy detachment of the bubbles. Similarly, for HT NiFeW catalysts, an almost linear curve confirms the increased hydrophilicity of the surface. Also, the current density increases with time for the HT catalysts, indicating the slow activation of the electroactive sites for mixed oxide/hydroxide phase catalysts. It is also evident in Figure 4, where the performance of the HT NiFeW catalysts increased after the stability test.

**Figure 5:**
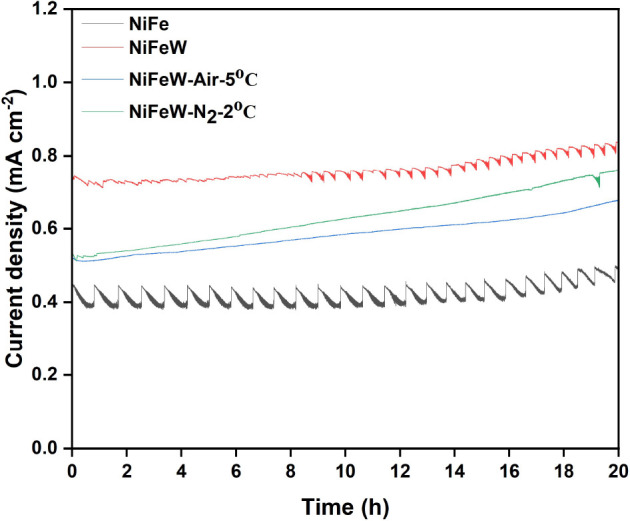
Stability test: The stability of the catalysts was appraised over 20 h at an overpotential of 300 mV (1.53 V vs RHE).

The electrochemical performance of the catalysts was examined using the EIS technique. Figure 6a,b displays the EIS data recorded before and after the stability test. In these data, there are two arcs at low and high frequencies. The presence of two arcs suggests that at least two relaxation processes are occurring in the system, each corresponding to different physical or chemical phenomenon with a distinct time constant. With ISGP, these data were analyzed to discern the relaxation processes.

**Figure 6:**
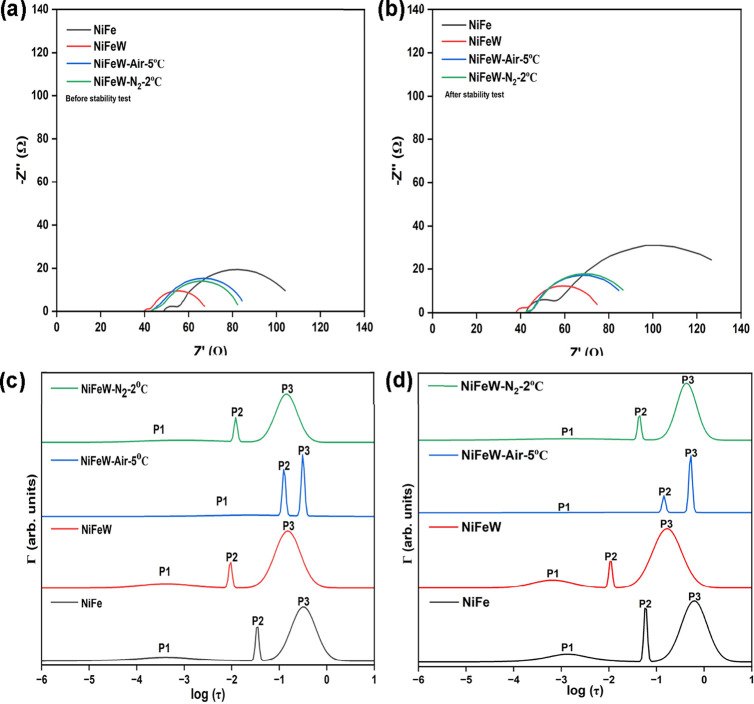
EIS was conducted both (a) before and (b) following the stability test at a frequency range of 0.1 to 10^5^ Hz at an overpotential of 300 mV. DFRT plots (not in scale): (c) before and (d) after the stability test.

The arc expands after the stability test, indicating an increase in the total polarization resistance of all of the catalysts. The resistance contributions of the different processes can be calculated from the ISGP results by multiplying the area under each peak by the normalization factor for the run. The latter is usually the maximum value of the real part of the impedance.

OER catalysts have three relaxation processes within the commonly measured frequency range (excluding solution resistance). These are related to P1, the active material; P2, charge transfer; and P3, the production rate of intermediates. The identification of the peaks’ origins is based on their characteristic frequencies and responses to changes under the system and the measurement conditions.^58^ The solution resistance can be found by a virtual peak at a very high frequency, which is out of the measured range. The increasing order of the relaxation times of the various processes is as follows: P1 < P2 < P3. Figure 6c,d presents the DFRT plots extracted from the EIS data by using ISGP. Figure 6c exhibits the DFRT plots for the catalysts before the stability test, and Figure 6d shows the DFRT plots for the catalysts after the stability test. From the DFRT peaks, the effective resistance and capacitance are determined. A comparative analysis of the DFRT plots before and after the stability test reveals a shift toward longer relaxation times across all catalysts, indicating a deceleration toward equilibrium. This suggests a retardation in reaction kinetics post-stability testing.

Before the stability test, the DFRT plots of catalysts with and without tungsten doping (Figure 6c) demonstrate distinct behaviors. Notably, the position of peak P1 remains unchanged. Conversely, peaks P2 and P3, which represent charge transfer and the production rate of intermediates, respectively, exhibit a shift to lower relaxation times in the presence of tungsten, suggesting faster charge transfer and rate of intermediate production. The peaks associated with the tungsten-doped catalyst that underwent HT in an inert atmosphere exhibit a slight shift compared to those of the tungsten-doped catalyst without HT. Peaks P1 and P3 demonstrate a shorter relaxation time, denoting accelerated processes. However, peak P2 shifts toward a high tau (τ) value, indicating a slowdown in the charge transfer process. Furthermore, all peaks from the heat-treated catalyst in the air shift toward higher relaxation times, reflecting slower processes. This behavior is likely due to a stable and comparatively slow-reacting oxide phase.

Figure 6d shows the DFRT plots after the stability test. The kinetics of the processes in the tungsten-doped catalysts are significantly enhanced compared to those of the undoped catalyst, as evidenced by the lower tau values of all peaks, which indicates reduced relaxation times. All peaks associated with the heat-treated catalysts exhibit a shift toward higher relaxation times compared to those of the tungsten-doped catalyst, indicating slower reaction kinetics. Hence, the tungsten-doped catalyst demonstrates a superior performance with faster process rates.

Table 5 presents the calculated resistances of all of the catalysts. Before and after the stability test, the undoped catalyst exhibits the highest total resistance, whereas the tungsten-doped catalyst exhibits the lowest. Lower effective resistances in the Faradaic peaks, P2 and P3, suggest that tungsten enhances the electrochemical efficiency of the catalyst. The DFRT peaks also enable the derivation of the respective capacitance values, of which the double-layer capacitance (*C*_dl_) is associated with the electrode–electrolyte interface. The double-layer capacitance is directly proportional to the electrochemically active surface area (ECSA) of the catalysts.^59^ The calculated double-layer capacitances (*C*_dl_) of the catalysts before/after the stability test are as follows: 8.35/6.69 mF for NiFe, 6.91/6.57 mF for NiFeW, 9.41/17.7 mF for NiFeW-Air-5 °C, and 5.29/14.9 mF for NiFeW-N_2_-2 °C. As observed, after the stability test, the *C*_dl_ of the NiFe catalyst has decreased, validating its deteriorated electrochemical performance. For the NiFeW catalyst, there were no significant changes in *C*_dl_ values, indicating no change in the active sites.^60^ Conversely, the catalysts subjected to HT exhibited an increase in their *C*_dl_ after the stability test, suggesting a rise in electroactive sites, which enhances catalytic activity, as also indicated from the LSV curves.

**Table 5:** Calculated Resistances of All the Catalysts

	**catalyst**	**P1 [Ω]**	**P2 [Ω]**	**P3 [Ω]**	**total resistance [Ω]**
before CA	NiFe	7.84	4.12	44.94	56.90
	NiFeW	2.93	1.35	22.58	26.86
	NiFeW-Air-5 °C	11.57	13.36	17.18	42.11
	NiFeW-N_2_-2 °C	5.23	2.29	31.31	38.83
after CA	NiFe	16.07	8.79	71.00	95.86
	NiFeW	5.37	1.64	29.58	36.59
	NiFeW-Air-5 °C	7.57	8.06	28.79	44.42
	NiFeW-N_2_-2 °C	4.74	2.93	39.10	46.77

The optimal catalyst ratio was tested in an anion exchange membrane (AEM) electrolyzer. The anode was composed of NiFeW deposited on Ni foam and the cathode of commercial Pt–Ru/C (Pt 20 wt %, Ru 10 wt % on graphitized carbon). The cell was activated by one cycle between 1.3 and 2.0 V, including a 10 min hold at 1.4 V. The polarization curve was recorded after activation. The NiFeW catalyst exhibits an excellent electrocatalytic performance. Figure 7 shows a relatively low open-circuit voltage (OCV) in the polarization curve of approximately 1.47 V, and it reaches very high current densities of 2.12 A cm^–2^ at ∼2 V. This suggests favorable OER kinetics and good conductivity under operational conditions. The high activity of the NiFeW catalyst can be attributed to the synergistic effect between nickel, iron, and tungsten. These features make NiFeW a promising material for scalable hydrogen production through alkaline or AEM electrolyzers.

**Figure 7:**
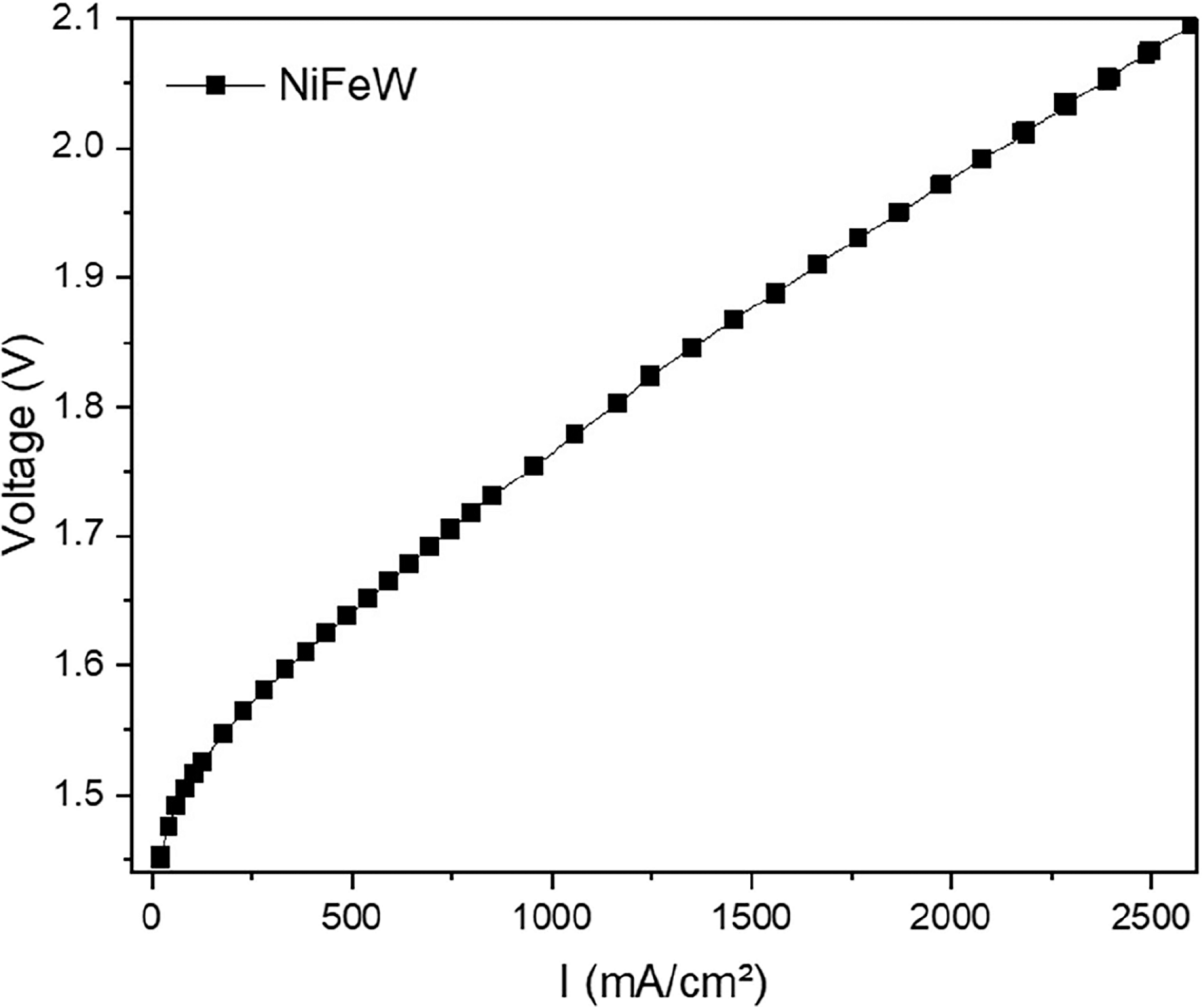
Polarization curve of an AEM electrolyzer consisting of an anode composed of 2 mg/cm^2^ NiFeW on Ni foam and a cathode composed of 0.5 mg cm^–2^ Pt–Ru/C-based cathode and a 40 μm PiperION AEM. The cell was operated at 70 °C with 1 M KOH flowing on both sides.

### 3.3 DFT Results

To gain further insight, we conducted DFT calculations to explore the impact of tungsten (W) doping on the OER. Specifically, we aimed to understand how W doping enhances the OER activity, to identify the specific sites where the OER occurs, and to understand how W doping reduces charge transfer resistance.

In this study, the overpotential is calculated using the reaction mechanism outlined in the Supporting Information (eqs 1–16 in the Supporting Information for the overpotential calculation). Initially, the metal slab adsorbs two water molecules on its two exposed metal sites, with one water molecule at each site. In the first deprotonation step, the adsorbed water molecule at the active site loses a proton, while the other adsorbed water molecule remains intact and acts as a spectator throughout the OER. This step forms the *OH reaction intermediate at the active site.

In the second deprotonation step, the *OH intermediate loses a proton, resulting in the formation of the *O intermediate at the active site. During the third deprotonation step, the *OOH intermediate is formed when a water molecule interacts with the *O intermediate. In the fourth step, the *OOH intermediate loses a proton, leading to the formation of the *OO at the active site. Finally, in the fifth step, the *OO species desorbs from the active site as molecular oxygen, allowing a water molecule to adsorb in its place.

As a result, molecular oxygen evolved on the catalyst surface, completing the cycle and returning to the starting point of the reaction. This process continues cyclically, consistently producing molecular oxygen. Figure 8 shows the reaction mechanism, along with the calculated overpotential.

**Figure 8:**
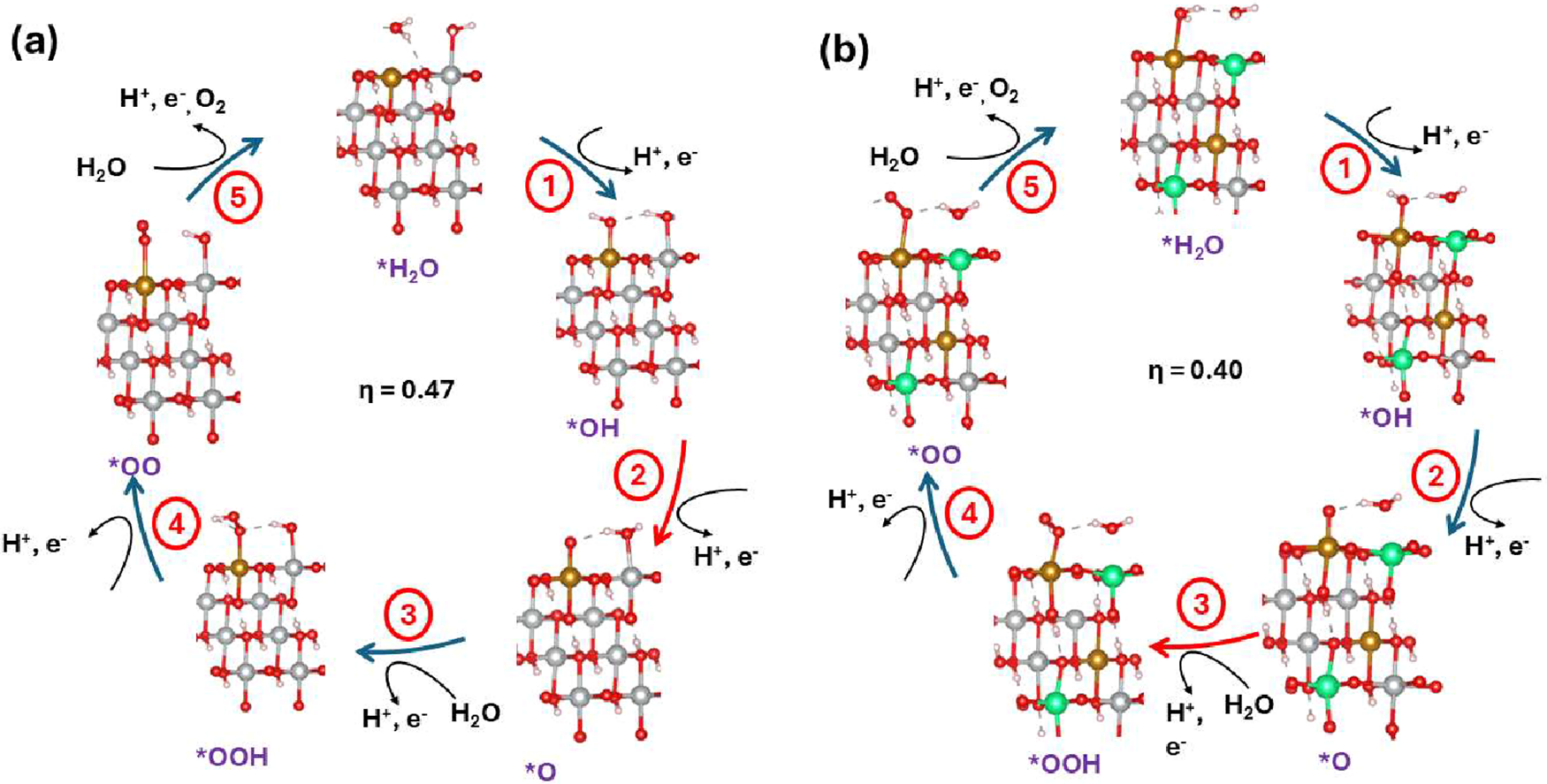
Reaction mechanism of the OER on (a) NiFeOOH and (b) NiFeWOOH with W and Fe on the top. Ions color code (Gold: Fe, Silver: Ni, Green: W, Red: O, and Pink: H). The potential-determining step is marked in red.

Step reaction energies of the OER are calculated and mentioned in Table 6, and also shown as step energy graphs in Figure 9. The potential-determining step is marked in bold in Table 6.

**Table 6:** OER Step Energies (1–5) (eV), Overpotential (η) (V), and Work Function (Φ) (eV) of These Oxy-Hydroxides^a^

**material**	**1**	**2**	**3**	**4**	**5**	**η**	**Φ**
NiFeOOH	1.38	**1.59**	1.25	0.38	–0.14	0.47	5.93
NiFeWOOH-**Fe**Ni	1.00	0.90	**2.22**	–0.63	0.98	1.10	5.20
NiFeWOOH-W**Fe**	0.81	1.33	**1.52**	0.35	0.45	0.40	5.59
NiFeWOOH-**W**Fe	0.87	0.51	**3.08**	–0.68	0.68	1.96	5.59
NiFeWOOH-Fe**Ni**	**2.28**	1.28	1.29	1.60	–1.97	1.16	5.26

a Material symbols are explained in Table 1 (Section 2).

**Figure 9:**
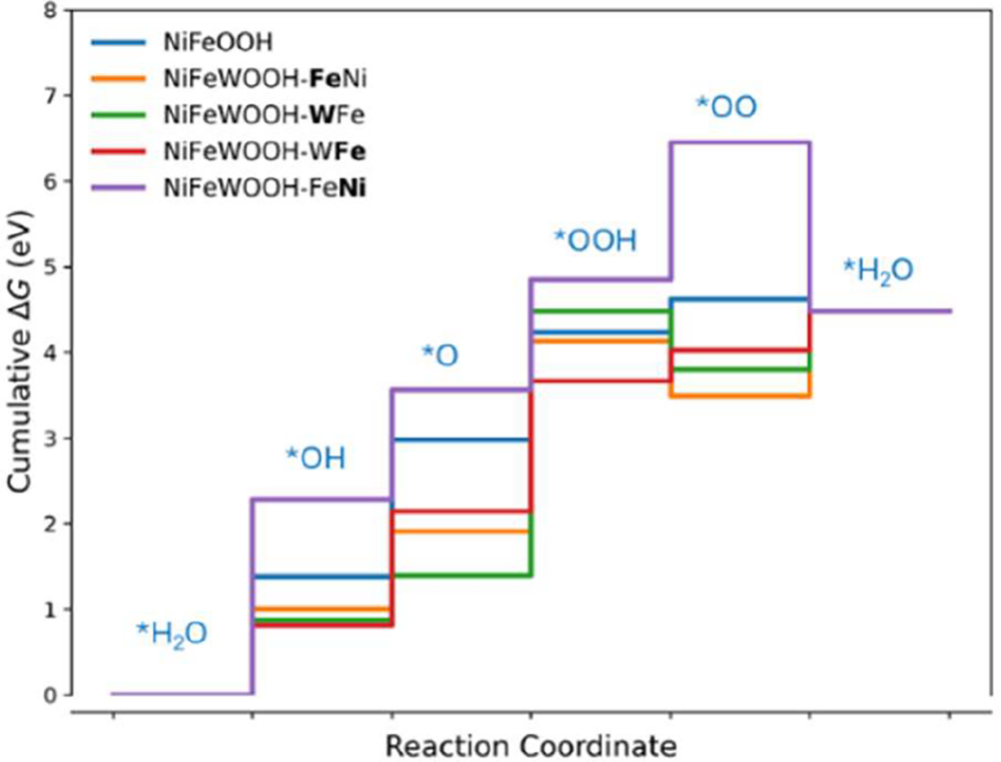
Cumulative free energy of the OER on various catalysts surfaces. These energies are calculated at pH zero and applied potential 0 V. Values at high pH and potentials are given in Table S4 in the Supporting Information.

For pure NiFeOOH (without any W) with Fe as the active site, the overpotential is 0.47 V and the potential-determining step is step 2 (*OH → *O). The calculated overpotential is consistent with previous results, where an overpotential between 0.42 and 0.57 V is reported.^61–64^ Also, the potential-determining step is the same as reported in the literature.^61^ Other studies have reported different overpotentials; however, these involve different structures/reaction mechanisms.^38,40,65,66^ For NiFeOOH with W (NiFeWOOH-W**Fe**), when W and Fe are at the top of the slab and Fe is the active site, the overpotential decreases to 0.40 V, and the potential-determining step changes to step 3 (*O → *OOH). In this study, other possible scenarios where Fe and Ni are at the top and Fe acting as the active site (NiFeWOOH-Ni**Fe**) and Ni acting as the active site (NiFeWOOH-**Ni**Fe), as well as the W and Fe at the top and W acting as the catalytic site (NiFeWOOH-**W**Fe), are also considered. Table 6 shows that, in all cases except NiFeWOOH-WFe, the overpotential is comparatively higher than that of NiFeOOH. Surprisingly, when W acts as the active site (NiFeWOOH-**W**Fe), the overpotential is very high (1.96 V).

Our overpotential calculations show that NiFeWOOH-W**Fe** exhibits the lowest overpotential (0.40 V) among all the materials studied, which is also lower than that of NiFeOOH (0.47 V). In NiFeOOH, the relatively weak adsorption of *O (4.09 eV) compared to *OH (2.49 eV) makes *OH → *O (step 2) energetically demanding and therefore the potential-determining step. Upon W doping (NiFeWOOH-W**Fe**), the *O intermediate is stabilized, with its adsorption free energy decreasing to 3.72 eV, while the *OOH adsorption free energy is only slightly reduced from 5.34 to 5.25 eV. As a result, the energetic barrier for the *OH → *O step decreases, whereas the adsorption free energy gap between *O and *OOH increases, leading to a shift of the potential-determining step from *OH → *O (step 2) in NiFeOOH to *O → *OOH (step 3) in NiFeWOOH-W**Fe**. This shift directly explains the observed overpotential trend (Table 6) and shows that W doping reduces the OER overpotential by selectively tuning the relative adsorption strengths of the key oxygen intermediates. For the adsorption free energies of all reactants and reaction intermediates, please refer to Table S3. Thus, W doping reduces the OER overpotential at the Fe active site and is beneficial for the oxygen evolution reaction. Other potential active sites, such as Ni and W, were also investigated but showed significantly higher overpotentials. For instance, when W acts as the active site, the overpotential reaches as high as 1.96 V, suggesting that W does not directly participate in the OER.

To further investigate the adsorption aspects of these materials, the d-band center of all these materials was calculated (see the Supporting Information) using the No̷rskov model in VASPKIT.^67,68^ The results show that W cannot directly participate in the OER, as its W 5d-states are located approximately 0.37–1.09 eV above the Fermi level, making it unable to form a covalent bond.^25^ Similar results of d-band center calculations were seen in the case of W doping in NiOOH in the study of Ghosh et al.^25^ The calculated d-band center also reveals that the d-band center of Fe is higher than that of Ni, and both lie below the Fermi level. The same trend can be observed in the DOS results (Figure S9), where W states are not present near the Fermi level but appear in the conduction band above the Fermi level. States near or slightly below the Fermi level belong to Ni and Fe (apart from oxygen). This explains the very high overpotential observed in the case of NiFeWOOH-**W**Fe when W is the active site.

Bader analysis and the magnetization data (see the Supporting Information) indicate that W is in the 6^+^ oxidation state, with around −2.82 e Bader charge and zero magnetization. Bader analysis also confirms that W donates its electrons to other metal ions (Fe and Ni). For example, in the case of Ni and Fe in NiFeOOH, the average Bader charges are approximately −1.27 and −1.74 e, respectively. After W doping, the average Bader charges on Ni and Fe increase to −1.23 and −1.68 e, respectively (increase/decrease in Bader charge means gain/loss of electrons). This shows the electron-donating nature of the W. A similar phenomenon is observed in the DOS graphs (see Figure S9), which shows the appearance of new electronic states for Fe and Ni near the Fermi level after W doping. This electron-donating behavior of W is also evident in the charge density difference analysis (Figure S10), where the charge from W accumulates on nearby Ni and Fe atoms.

The work function of all of the materials was calculated when all of the metal sites were covered by water and tabulated in Table 6. It shows that when W is doped into the material, the work function decreases to 5.20 eV (in the case of NiFeWOOH-**Fe**Ni) compared to 5.93 eV for NiFeOOH (without any W). The general trend here indicates that W doping significantly decreases the work function. A decrease in the work function suggests an upward shift in the Fermi level of the material, indicating N-type doping.^69,70^ This aligns with other results (Bader charge, charge density difference, and DOS), suggesting that W donates electrons. N-type doping increases the electron concentration in the system, increasing electronic conductivity,^71,72^ which consequently leads to lower charge transfer resistance.^73,74^ The decreased work function of the material is also attributed to efficient charge transfer during the electrochemical reaction.^75,76^ Thus, the reduced work function and the lower overpotential of NiFeWOOH-W**Fe** lead to a decreased charge transfer resistance observed in EIS.

In conclusion, the DFT results show that W doping lowers the overpotential at the Fe active site. The d-band center analysis indicates that the W site itself is not favorable for the OER. The DOS, Bader charge analysis, charge density difference, and work function consistently show the electron-donating nature of W. The calculations further reveal a decrease in work function upon W incorporation. This agrees well with our experimental studies, where W-doped catalysts exhibit improved OER activity and reduced charge transfer resistance.

## 4 Conclusions

This research highlights the significant potential of solid solution/doping of tungsten into nickel–iron hydroxide catalysts to enhance the efficiency of OER catalysis, a critical step in water splitting technologies. Adding tungsten to the NiFe hydroxide structure is aimed at improving the catalytic activity and stability. Techniques such as LSV, XPS, EIS, and DFRT analyses provided insights into the electrochemical phenomena influencing the OER activity. These analyses confirmed that tungsten reduces the overpotential and enhances the kinetics of water oxidation by decreasing the effective resistance of the catalyst. XRD results indicated a solubility limit of 27% for tungsten doping and demonstrated a significant lattice expansion in the c direction by ∼30%, influencing the electronic environment and leading to decreased binding energies. Additionally, the effects of various HTs under air and nitrogen atmospheres significantly affected the physical and chemical properties of the catalysts. Nitrogen treatment results in mixed phases in the catalyst, both hydroxide and oxide, with the oxide phase typically requiring more time to become active, as confirmed by the stability tests over a 20 h period. The test also suggested that the catalysts might reach a stable activity level if allowed to run longer, potentially enhancing their applicability in commercial electrolysis systems. DFT calculations show a decrease in overpotential to 0.40 V when Fe and W are present in the top layer, with the reaction occurring at the Fe site. The d-band center analysis indicates that the tungsten cannot function as the catalytic site, as its d-band centers are positioned approximately 0.37–1.09 eV above the Fermi level. Also, DFT calculations reveal a significant decrease in the work function to 5.20 eV and an upward shift in the Fermi level due to W doping, resulting in reduced charge transfer resistance. In summary, the improved electrocatalytic performance, decreased effective resistances, and enhanced stability introduced by the tungsten solid solution in NiFe hydroxides offer a promising pathway for future research and development. Since this is a ternary metal system, further work is needed to find the “global” solubility limit, as well as the Ni/Fe/W ratios that give the best catalytic performance.

## Supplementary Material





## Notes

LSV comparison curves for the heat-treated catalysts, the differential thermogravimetric analysis (dTG) and differential thermal analysis (DTA) curves of NiFeW, the XRD spectra of several other synthesized materials, nitrogen adsorption–desorption curves, and BJH desorption cumulative pore volume plots of the catalysts. The SI also includes more details on the computational and Bader charge data (PDF)
Structural information and magnetization data (ZIP)
