# Supplementary material for: Toward Efficient Hydrogen Production: Impact of Solid Solution of Tungsten on Nickel–Iron Hydroxide OER Catalysts
Source: ACS Catal. 2026 Feb 6;16(5):4449–62. doi: 10.1021/acscatal.5c07061 (PMC12976961; doi:10.1021/acscatal.5c07061)
Supplement: Supplementary file 1 [file cs5c07061_si_001.pdf]

Supporting Information

Towards Efficient Hydrogen Production: Impact of Solid Solution of Tungsten on Nickel-Iron Hydroxide OER Catalysts

Lamea Abbas[a], Lakhanlal[b], Sourav Bhowmick[a], Rawnaq Batheesh[c], Lior Elbaz[c], Maytal Caspary Toroker[b,d,e*], and Yoed Tsur [a,e*]

[a] The Wolfson Department of Chemical Engineering, Technion- Israel Institute of Technology, Haifa 3200003, Israel.

[b] Department of Materials Science and Engineering, Technion- Israel Institute of Technology, Haifa 3200003, Israel

[c] Department of Chemistry, Bar-Ilan Center for Nanotechnology and Advanced Materials, Bar-Ilan University, Ramat-Gan 5290002, Israel

[d] Resnick Sustainability Center for catalysis, Technion – Israel Institute of Technology, Haifa 3200003, Israel

[e] The Nancy and Stephen Grand Technion Energy Program, Technion- Israel Institute of Technology, Haifa 3200003, Israel.

Corresponding Author Information:

*
Prof. Maytal Caspary Toroker,
Department of Materials Science and Engineering,
Technion-Israel Institute of Technology, Haifa-3200003, Israel
Phone: +972-4-829-4298
E-mail: maytalc@technion.ac.il
ORCID ID: https://orcid.org/0000-0003-1449-2977

*

Prof. Yoed Tsur,
The Wolfson Department of Chemical Engineering,
Technion- Israel Institute of Technology, Haifa 3200003, Israel
Phone: +972-4-8293586
E-mail: tsur@technion.ac.il

The curves S1-S3 compare the linear sweep voltammetry (LSV) of the heat-treated catalysts under various conditions. Depending on these comparing curves, the catalyst that demonstrated the most effective electrochemical performance was chosen (400℃ with 5℃/min).

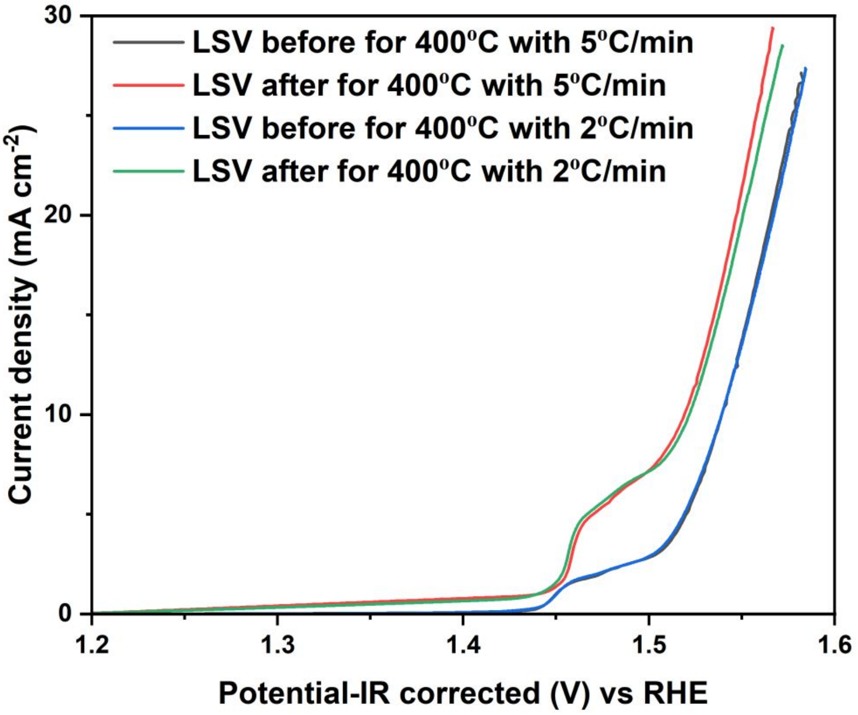

Figure S1. Polarization curves of the synthesized catalysts

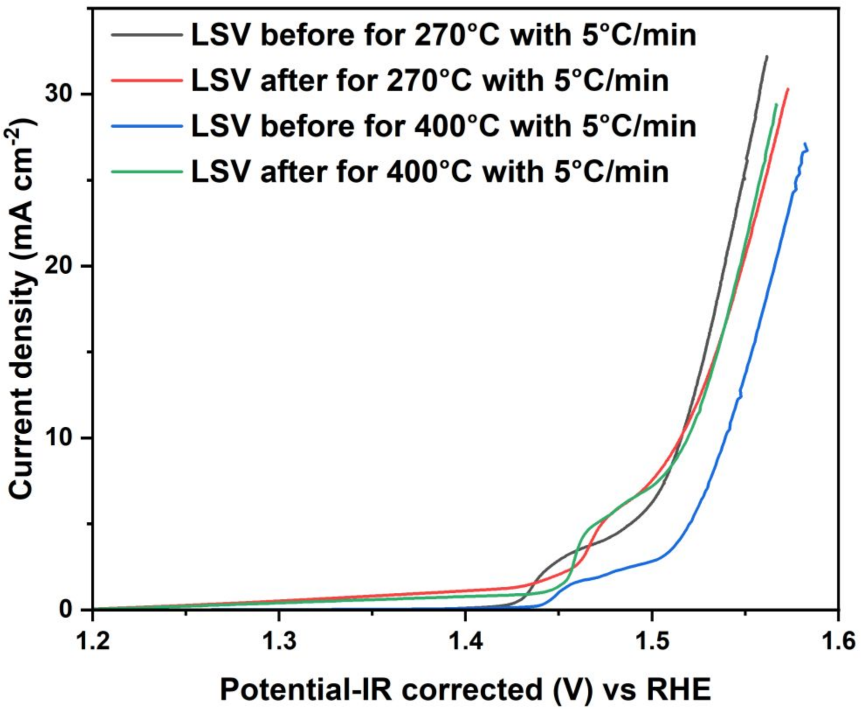

Figure S2. Polarization curves of the synthesized catalysts

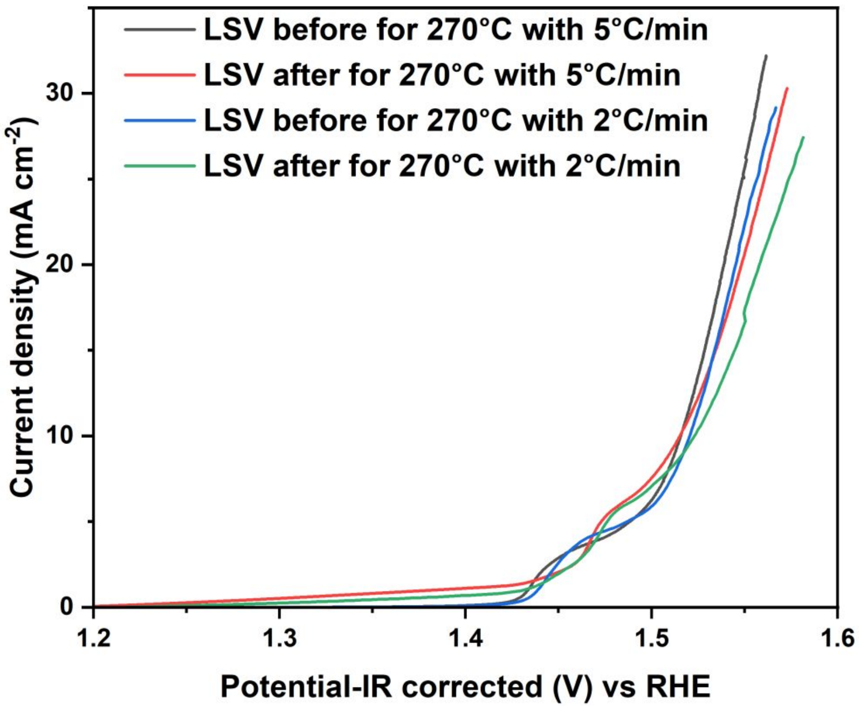

Figure S3. Polarization curves of the synthesized catalysts

Figure S4 displays the differential thermogravimetric analysis (dTG) and differential thermal analysis (DTA) curves of NiFeW confirming the weight loss resulting from an endothermic reaction.

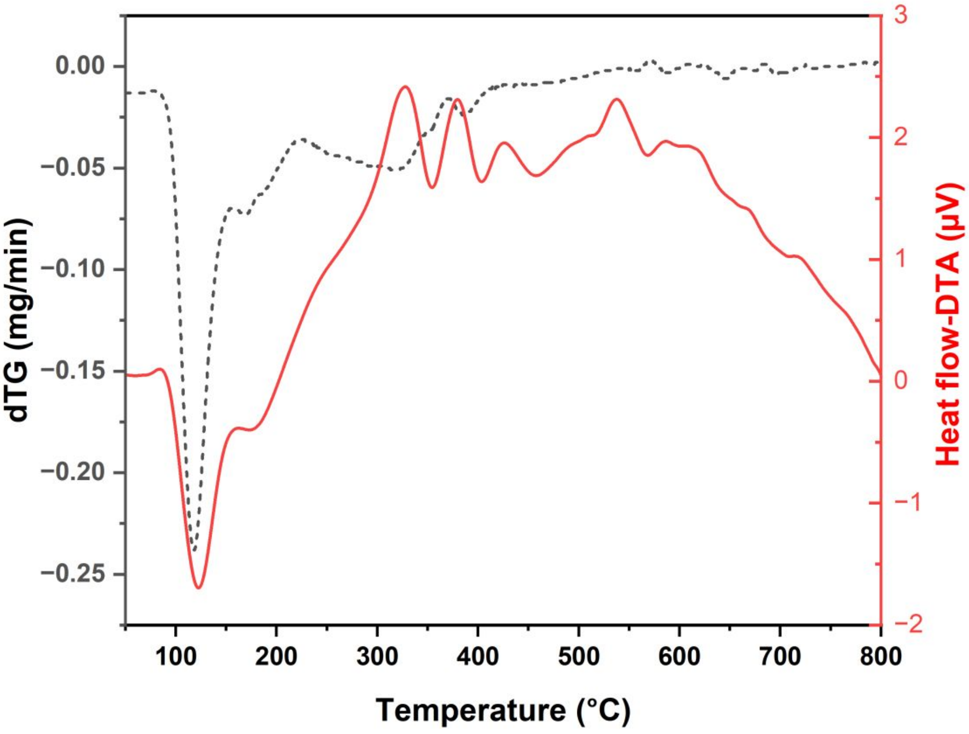

Figure S4. dTG and DTA curves of NiFeW

The XRD spectra of several other synthesized materials are shown in Figure S5. In some catalysts, peaks of impurities were detected. This allows us to assess the solubility limit of tungsten in the system, which is around 27%.

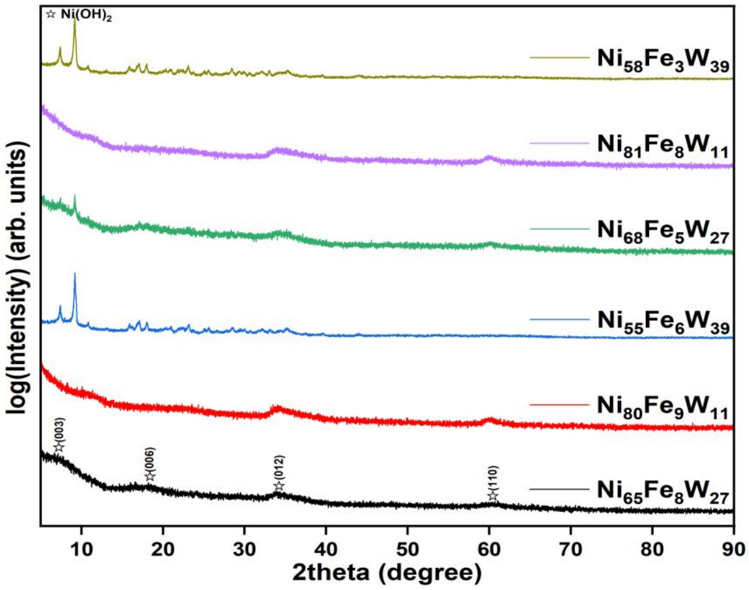

Figure S5. XRD pattern of the synthesized catalysts

Referring to figures S6 and S7 for nitrogen adsorption-desorption curves and BJH desorption cumulative pore volume plots of the catalysts, respectively, the surface area, total pore volume, and average pore diameter of the catalysts were determined.

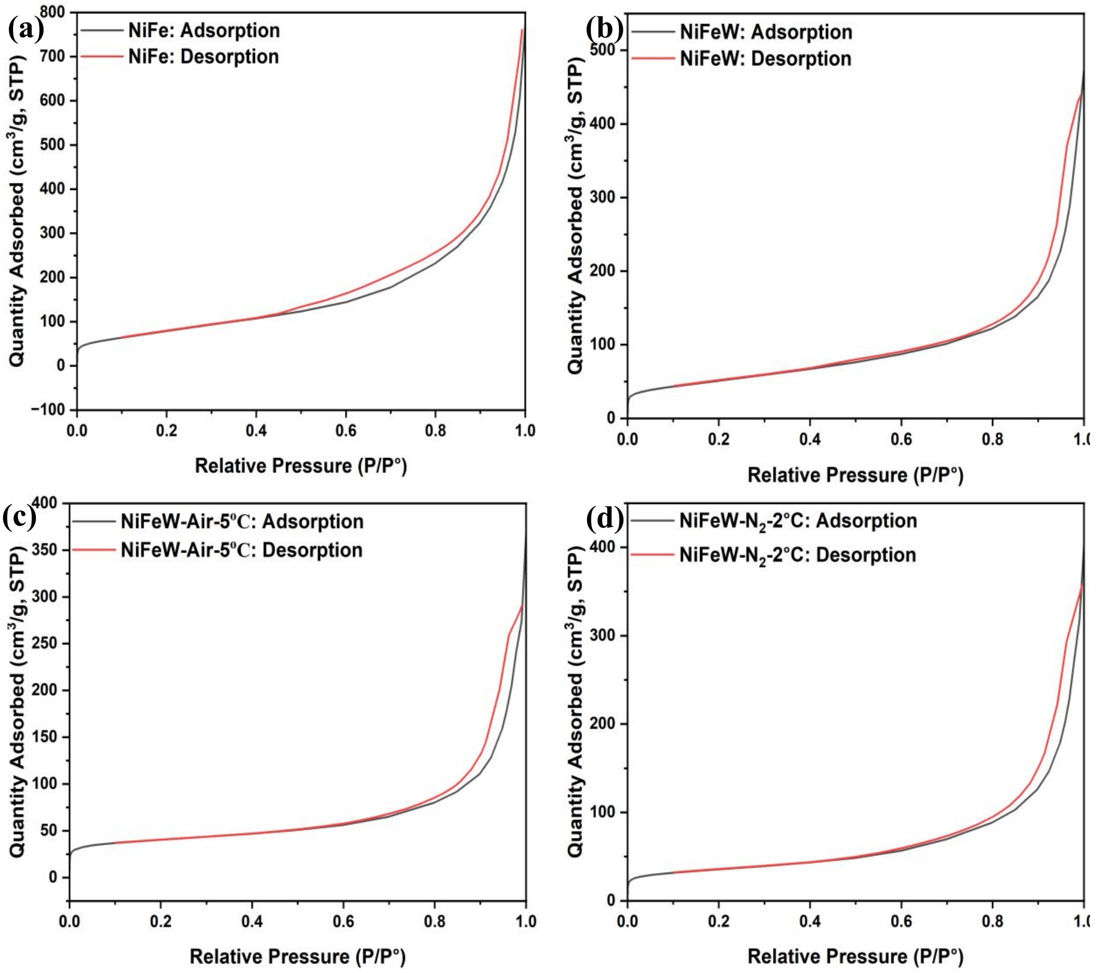

Figure S6. Nitrogen adsorption-desorption curves for (a) NiFe, (b) NiFeW, (c) NiFeW-Air-5℃ and (d) NiFeW-N_2-2℃

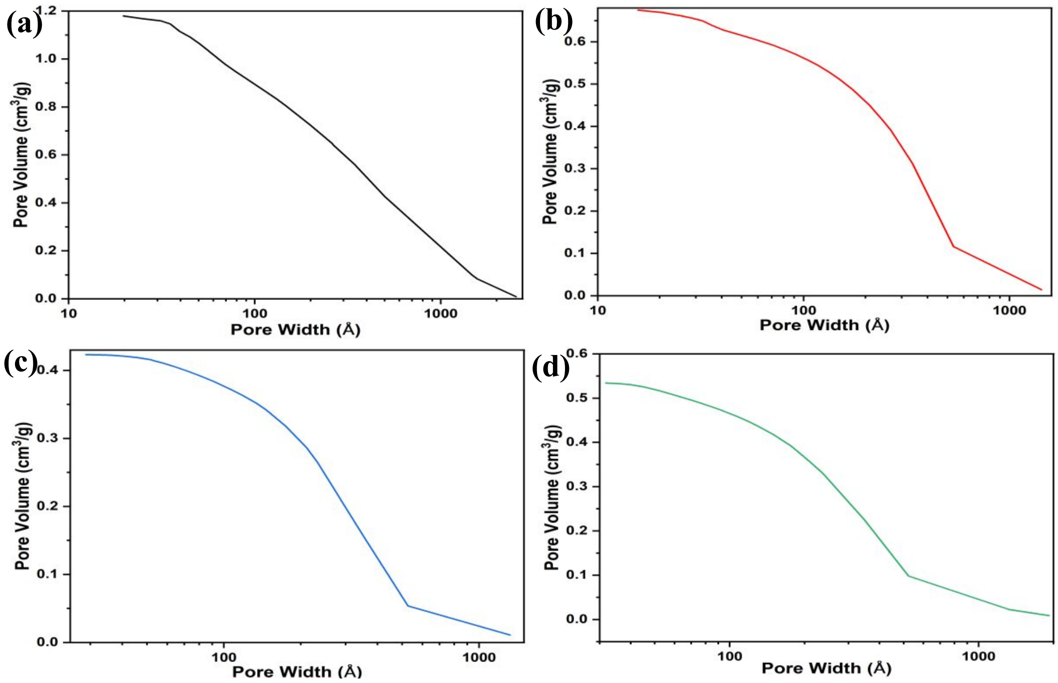

Figure S7. BJH Desorption Cumulative Pore Volume plots for (a) NiFe, (b) NiFeW, (c) NiFeW-Air-5℃ and (d) NiFeW-N_2-2℃

Comparing the LSV curves of all the as-synthesized catalysts is presented in Figure S8. The best catalyst was chosen depending on these curves, which is $Ni_{65}Fe_8W_{27}$, demonstrating the best electrochemical performance.

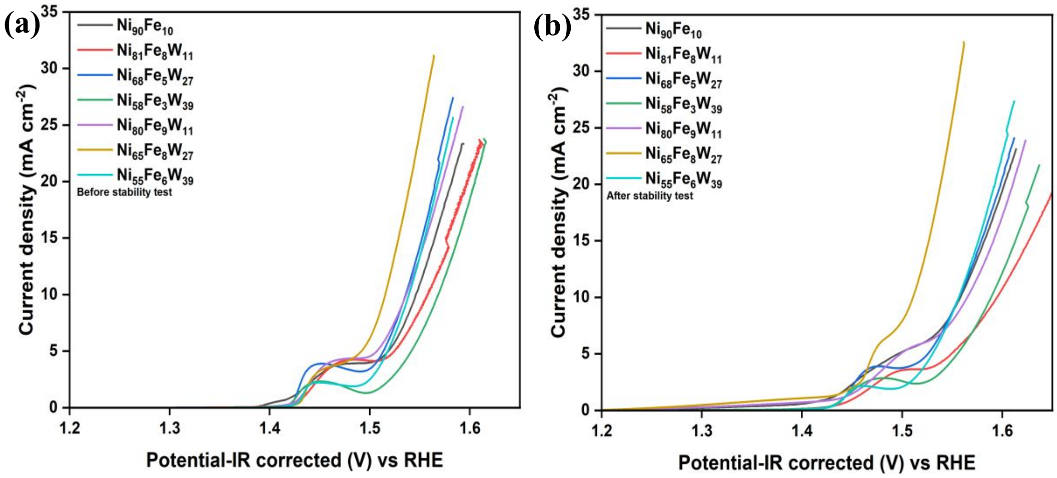

Figure S8. Polarization curves of the synthesized catalysts (a) before and (b) after the stability test

Overpotential calculation

For OER, overpotential is calculated for all the above mentioned materials in the table. In the slab model, at the top, there are two exposed metal atoms. Depending upon the material, the exposed atoms could be from Ni, Fe, and W. Also, the OER happens only on a single metal site, and another site always have an adsorbed water molecule (see **Figure 10** for the reaction mechanism). The reaction steps are:

Step 1: $Catalyst_{\_H2O} \rightarrow Catalyst_{\_OH} + (H^+ + e^-)$ (1)

Step 2: $Catalyst_{\_OH} \rightarrow Catalyst_{\_O} + (H^+ + e^-)$ (2)

Step 3: $Catalyst_{\_O} + H_2O \rightarrow Catalyst_{\_OOH} + (H^+ + e^-)$ (3)

Step 4: $Catalyst_{\_OOH} \rightarrow Catalyst_{\_OO} + (H^+ + e^-)$ (4)

Step 5: $Catalyst_{\_OO} + H_2O \rightarrow Catalyst_{\_H2O} + O_2$ (5)

The adsorption energy of the reaction intermediates is calculated using the equations given below:

$$\Delta G_{H_2O} = E^*_{H_2O} - E_{slab} - 2E_{H_2O} \quad (6)$$

$$\Delta G_{OH} = E^*_{OH} - E_{slab} - 2E_{H_2O} + 1/2\ E_{H_2} \quad (7)$$

$$\Delta G_{O} = E^*_{O} - E_{slab} - 2E_{H_2O} + E_{H_2} \quad (8)$$

$$\Delta G_{OOH} = E^*_{OOH} - E_{slab} - 3E_{H_2O} + 3/2E_{H_2} \quad (9)$$

$$\Delta G_{OO} = E^*_{OOH} - E_{slab} - 3E_{H_2O} + 2E_{H_2} \quad (10)$$

The reaction energies of the different reaction steps were calculated using equations below:

$$\Delta G_1 = E^*_{OH} - E^*_{H_2O} + 1/2\ E_{H_2} + 1/2\ (ZPE - T\cdot\Delta S)_{H_2} \quad (11)$$

$$\Delta G_2 = E^*_{O} - E^*_{OH} + 1/2\ E_{H_2} + 1/2\ (ZPE - T\cdot\Delta S)_{H_2} \quad (12)$$

$$\Delta G_3 = E^*_{OOH} - E^*_{O} + 1/2\ E_{H_2} - E_{H_2O} + 1/2\ (ZPE - T\cdot\Delta S)_{H_2} - (ZPE - T\cdot\Delta S)_{H_2O} \quad (13)$$

$$\Delta G_4 = E^*_{OO} - E^*_{OOH} + 1/2\ E_{H_2} + 1/2\ (ZPE - T\cdot\Delta S)_{H_2} \quad (14)$$

$\Delta G_5 = E^*_{H_2O} - E^*_{OO} + E_{O_2} - E_{H_2O} + (ZPE - T\cdot\Delta S)_{O_2} - (ZPE - T\cdot\Delta S)_{H_2O}$ (15)

Where E^*_x is the DFT energy of the reaction intermediates that includes the ZPE and T·ΔS correction. E_x is the energy of the gaseous molecules, ZPE and TS are the respective zero point and entropy correction.

Table S1 lists the DFT calculated energy of the empty slab along with the adsorbed reactant (H_2O) and the various adsorbed reaction intermediates. **Table S2** shows the ZPE-TΔS values for the adsorbed reactant and the reaction intermediates. For ZPE and TΔS calculation, all the atoms of the slab were not allowed to move. Only the adsorbed reactant or the reaction intermediates were allowed to vibrate. The ZPE and TΔS values were calculated using the VASPKIT, and the method used was specified in the VASPKIT user manual. Using the data of **Table S1** and **S2**, the adsorption energy (Table **S3**) is calculated followed by the calculation of step reaction energies. Overpotential is calculated as the difference between highest reaction energy among all reaction steps ($\max(\Delta G_i)$) and the DFT calculated $E^o_{ox/water}$ (1.12 V).

$$\eta = \max(\Delta G_i) - 1.12 \quad (16)$$

The step with highest reaction energy ($\max(\Delta G_i)$) is also known as potential determining step (PDS) and marked in bold in **table 5**.

Table S1: DFT calculated energy (eV)

**Material**	**Slab**	**$*H_2O$**	***OH**	***O**	***OOH**	***OO**
NiFeOOH	-230.99	-260.59	-255.32	-249.81	-259.98	-255.36
NiFeWOOH-**Fe**Ni	-286.95	-315.71	-310.81	-305.92	-315.18	-311.53
NiFeWOOH-W**Fe**	-287.12	-316.33	-311.62	-306.30	-316.28	-311.74
NiFeWOOH-**W**Fe	-287.12	-316.33	-311.61	-307.07	-315.41	-311.95
NiFeWOOH-Fe**Ni**	-286.84	-316.44	-310.28	-304.94	-315.14	-309.37

Table S2: ZPE-TΔS correction (eV)

**Material**	**Slab**	**$*H_2O$**	***OH**	***O**	***OOH**	***OO**
NiFeOOH	0	2.11	1.65	1.17	1.72	0.92
NiFeWOOH-**Fe**Ni	0	2.19	1.72	1.17	1.79	0.93
NiFeWOOH-W**Fe**	0	2.19	1.72	1.16	1.8	1.04
NiFeWOOH-**W**Fe	0	2.19	1.78	1.18	1.74	1.03
NiFeWOOH-Fe**Ni**	0	2.16	1.71	1.08	1.7	0.96

Table S3: Adsorption free energy (eV)

**Material**	**H_2O**	**OH**	**O**	**OOH**	**OO**
NiFeOOH	1.11	2.49	4.09	5.34	5.73
NiFeWOOH-**Fe**Ni	2.03	3.03	3.94	6.17	5.53
NiFeWOOH-W**Fe**	1.58	2.39	3.72	5.25	5.60
NiFeWOOH-**W**Fe	1.58	2.45	2.97	6.06	5.38
NiFeWOOH-Fe**Ni**	1.15	3.43	4.71	6.00	7.60

Effect of pH and applied voltage:

The pH as well as the applied voltage (V) affect the step energies with electron transfer as follows:

$$\Delta G_i(pH, V) = \Delta G_i - eV - K_B T \cdot ln10 \cdot pH \quad (17)$$

The pH (14) and voltage (1 V) dependent step energies are provided in the table S4. Step number five does not involve electron/proton transfer and thus remains pH and voltage-independent. The method used here for the calculation is already used and described in the literature[1,2].

Table S4: pH and potential dependent OER step energies (1-5) (eV) of these oxy-hydroxides

**Material**	**1**	**2**	**3**	**4**	**5**
NiFeOOH	-0.45	-0.23	-0.57	-1.44	-0.14
NiFeWOOH-**Fe**Ni	-0.83	-0.92	0.40	-2.47	0.98
NiFeWOOH-W**Fe**	-1.02	-0.49	-0.30	-1.47	0.46
NiFeWOOH-**W**Fe	-0.95	-1.31	1.26	-2.51	0.68
NiFeWOOH-Fe**Ni**	0.45	-0.55	-0.54	-0.23	-1.97

Bader charge and magnetization data:

Bader charge data of slab model of the NiFeWOOH-W**Fe** and NiFeOOH is given below in the table. For other material's as well as their other reaction intermediate's bader charge and magnetization data check the excel sheet provided in the zipped folder.

Table S5: Bader charge and magnetization data

NiFeWOOH-W**Fe**	**Ions**	**Bader Charge**	**Magnetization**	NiFeOOH/slab	**Ions**	**Bader Charge**	**Magnetization**
	H	-0.627378	0.003		H	-0.63558	0.004
	H	-0.631359	0.006		H	-0.64094	0.002
	H	-0.644979	0.005		H	-0.633001	0.002
	H	-0.630535	0.002		H	-0.635497	0.002
	H	-0.626329	0.002		H	-0.634742	-0.001
	H	-0.641617	0.005		H	-0.630223	0.004
	H	-0.650461	0.004		H	-0.625601	0.002
	H	-0.639437	0.005		H	-0.64437	0.002
	H	-0.655421	0.004		H	-0.635603	0.002
	H	-0.637684	0.001		H	-0.633446	-0.001
	O	1.057791	0.143		O	0.816815	0.043
	O	1.01363	0.073		O	0.724977	-0.224
	O	1.203006	0.122		O	0.830134	0.01
	O	1.158725	0.156		O	1.117973	0.026
	O	1.072016	0.074		O	0.828103	-0.065
	O	1.062519	0.145		O	1.091247	0.066
	O	1.185959	0.097		O	0.823597	0.031
	O	1.054219	0.041		O	1.027827	-0.076
	O	1.187795	0.176		O	0.846125	0.188
	O	1.217929	0.162		O	1.063097	0.046
	O	1.096725	0.071		O	1.097287	0.028
	O	1.054293	0.072		O	0.771359	-0.099
	O	1.048983	0.14		O	0.809477	0.042
	O	1.044335	0.063		O	0.745118	-0.215
	O	1.222448	0.147		O	0.803862	-0.025
	O	1.219896	0.145		O	1.105815	0.021
	O	1.103689	0.078		O	0.832137	-0.054
	O	1.068082	0.142		O	1.099599	0.062
	O	1.047325	0.062		O	0.818408	0.052
	O	1.18218	0.031		O	1.028911	-0.076
	O	1.027852	0.056		O	0.844034	0.03
	O	1.205476	0.163		O	1.078693	0.05
	O	1.245915	0.116		O	1.092846	0.026
	O	1.202295	0.087		O	0.768137	-0.099
	NI	-1.235528	1.778		NI	-1.233713	1.76
	NI	-1.242243	1.784		NI	-1.298231	0.147
	NI	-1.225565	1.763		NI	-1.252365	1.745
	NI	-1.258381	1.772		NI	-1.298396	1.116
	NI	-1.230982	1.778		NI	-1.293334	0.199
	NI	-1.236163	1.773		NI	-1.255485	1.344
	NI	-1.227252	1.762		NI	-1.294221	0.119
	FE	-1.789635	4.27		NI	-1.257531	1.741
	FE	-1.707907	4.165		NI	-1.294552	1.115
	W	-2.796107	0.017		NI	-1.29367	0.18
	W	-2.883589	0.05		NI	-1.279531	1.159
	W	-2.788637	0.016		FE	-1.68956	3.536

The average Bader charge of Ni, Fe, W, O, and H is given below in the table for the above shown two materials:

Table S6: Average Bader charge on Ions

Ions	NiFeOOH	NiFeWOOH-W**Fe**
Ni	-1.27	-1.23
Fe	-1.74	-1.68
W	-2.82	
O	1.12	0.91
H	-0.63	-0.63

The average Bader charge of -2.82 and close to zero magnetization on W suggests 6+ oxidation state.

Density of states results:

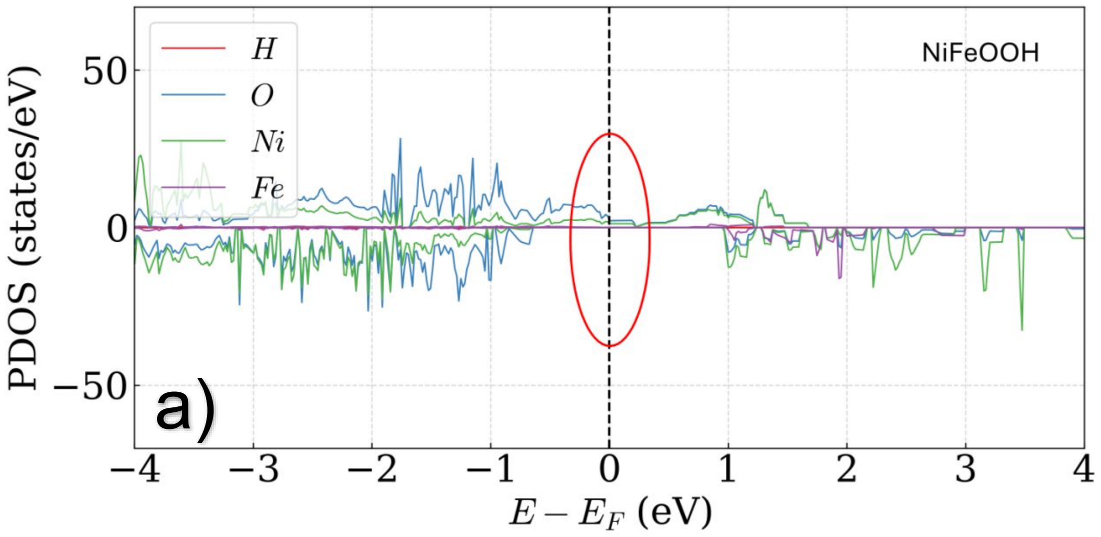

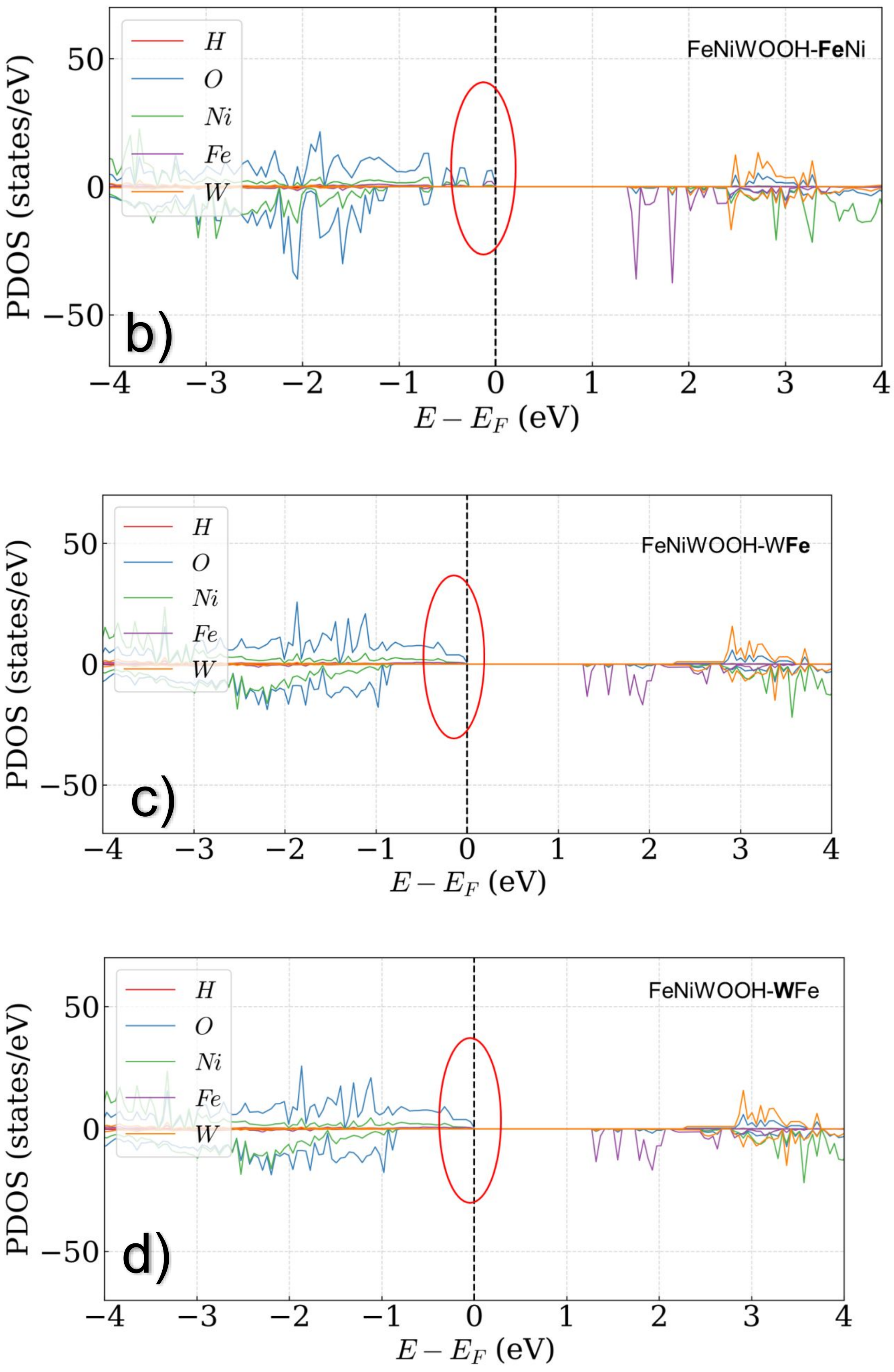

H
O
Ni
Fe
W
FeNiWOOH-**Fe**Ni
PDOS (states/eV)
b)
E − E_F (eV)
FeNiWOOH-W**Fe**
c)
FeNiWOOH-**W**Fe
d)

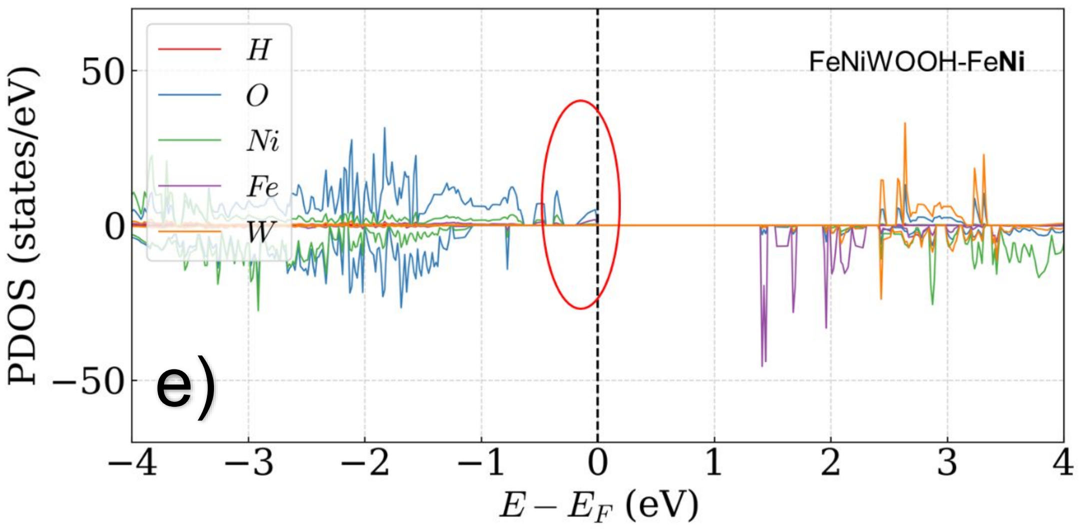

Figure S9: Density of state plots of catalysts. In the red circle, highlights new states formed due to W doping

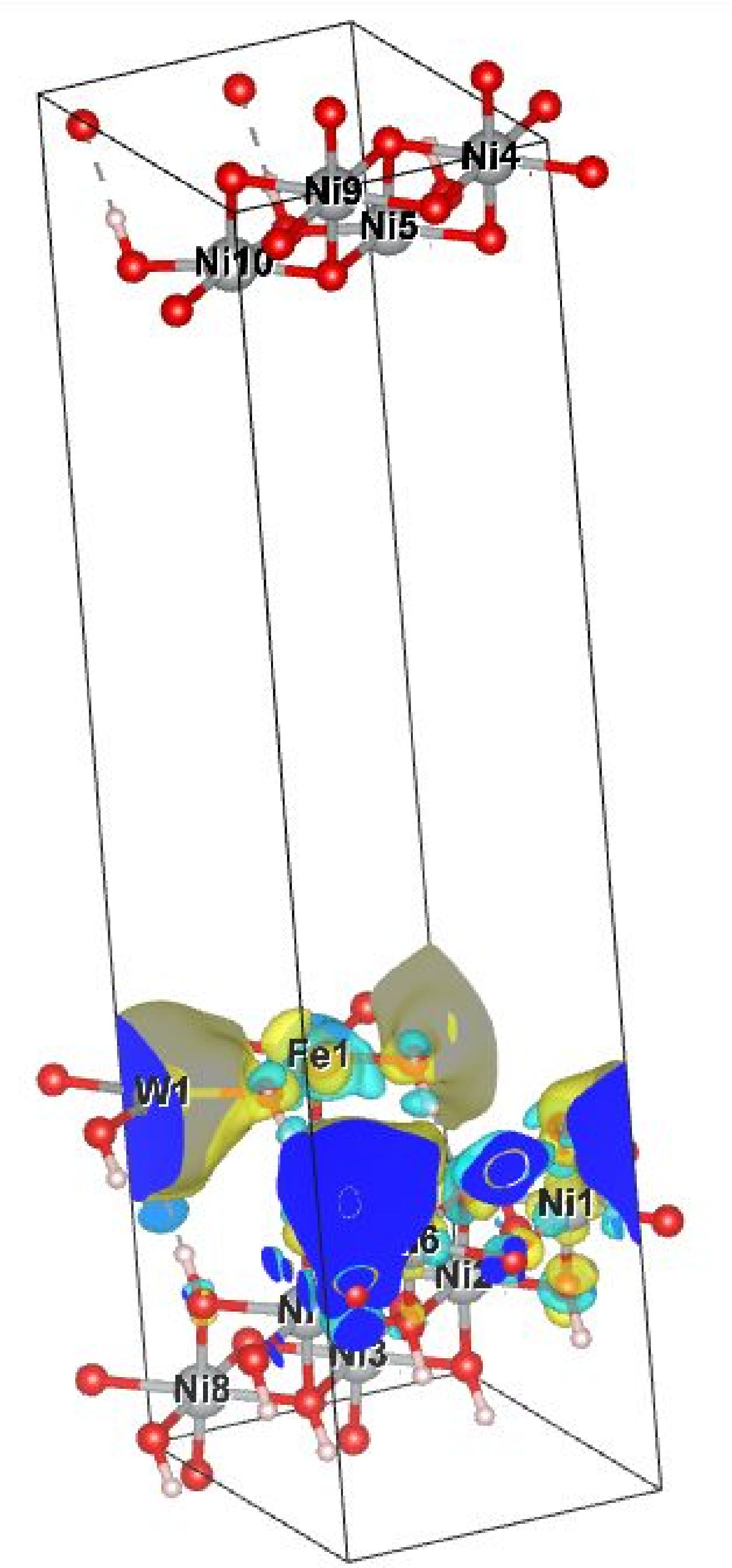

Figure S10: The charge density difference when one of the Ni in NiFeOOH gets replaced with the W (yellow: charge accumulation, cyan: charge depletion). It shows how charge (electrons) gets accumulated around Fe and Ni after W doping.

Work function calculation

Work function (Φ) of these materials are also calculated using the following equation.

$$\Phi = \phi_\infty - \mu \tag{18}$$

In the above equation, ϕ_∞ is the vacuum potential and μ is the chemical potential of the material.

The work function is given in table 5 of the main paper.

d-band Center calculation

d-band center (ϵ_d) is calculated using the VASPKIT software as the weighted average of the d-states.

$$\epsilon_d = \frac{\int_{-\infty}^{+\infty} \epsilon D(\epsilon) d\epsilon}{\int_{-\infty}^{+\infty} D(\epsilon) d\epsilon} \tag{19}$$

Here, the $D(\epsilon)$ is the number of d states per unit energy and $d\epsilon$ is the small differential energy. This formula has been used to calculate the d-band center in many literature[3,4].

The d-band center of the slab model of the NiFeWOOH-W**Fe** is shown below in **Table S7**.

Table S7: d-band center of individual metal ions in NiFeWOOH-W**Fe**

Elements	d-band center
NI	-2.64
NI	-3.254
NI	-3.13
NI	-2.691
NI	-2.807
NI	-2.796
NI	-2.621
FE	-1.891
FE	-2.668
W	0.993
W	0.791
W	0.376

For d-band center of other materials, please check the zipped folder.

Zipped folder description

The input files (INCAR), optimized structure files (CONTCAR), and bader.pqr is provided in a zipped folder for all the reaction intermediates. The d-band centre of the slab is their respective folder. Also an excel sheet is provided with the bader charge and magnetization data of all materials inside the zipped folder.

Table S8: Zipped folder details

**Computational Material**	**Code**	**Top layer atoms**	**Active site**	**Folder Name**
$Ni_{0.92}Fe_{0.08}OOH$	NiFeOOH	Ni, Fe	Fe	NiFeOOH
$Ni_{0.59}Fe_{0.16}W_{0.25}OOH$	NiFeWOOH-**Fe**Ni	Ni, Fe	Fe	NiFeWOOH-FeNi-active_Fe
$Ni_{0.59}Fe_{0.16}W_{0.25}OOH$	NiFeWOOH-W**Fe**	W, Fe	Fe	NiFeWOOH-WFe-active_Fe
$Ni_{0.59}Fe_{0.16}W_{0.25}OOH$	NiFeWOOH-**W**Fe	W, Fe	W	NiFeWOOH-WFe-active_W
$Ni_{0.59}Fe_{0.16}W_{0.25}OOH$	NiFeWOOH-Fe**Ni**	Fe, Ni	Ni	NiFeWOOH-FeNi-active_Fe

The energy of all optimized structures:

The energies of their respective structure of all materials are given below in eV.

Table S9: Energy of all structures

**Material**	**Slab**	**H_2O**	**OH**	**O**	**OOH**	**OO**
FeNiWOOH-**Fe**Ni	-286.95791578	-315.71225346	-310.81188985	-305.92223213	-315.18776434	-311.53614466
NiFeOOH	-230.99992158	-260.59535373	-255.32279217	-249.81797626	-259.98025350	-255.36336896
FeNiWOOH-W**Fe**	-287.12931241	-316.33532169	-311.62517601	-306.30107264	-316.28619789	-311.74231125
FeNiWOOH-**W**Fe	-287.12931241	-316.33532169	-311.61952026	-307.07013628	-315.41521641	-311.95691185
FeNiWOOH-Fe**Ni**	-286.84608180	-316.44998413	-310.28958410	-304.94852185	-315.14836314	-309.37683906

References:

1. Lakhanlal, Rimon O, Moschkowitsch W, Taguri GC, Elbaz L, Caspary Toroker M. Effect of heat treatment on improving OER activity of NiFeOOH based aerogels: A combined experimental and theoretical study. *Molecular Catalysis*. 2024;561:114164. doi:https://doi.org/10.1016/j.mcat.2024.114164

2. Liao P, Keith JA, Carter EA. Water Oxidation on Pure and Doped Hematite (0001) Surfaces: Prediction of Co and Ni as Effective Dopants for Electrocatalysis. *J Am Chem Soc*. 2012;134(32):13296-13309. doi:10.1021/ja301567f

3. Zhang Y, Xu J, Ding Y, Wang C. Tuning the d-band center enables nickel-iron phosphide nanoprisms as efficient electrocatalyst towards oxygen evolution. *Int J Hydrogen Energy*. 2020;45(35):17388-17397. doi:10.1016/J.IJHYDENE.2020.04.213

4. Qiao Z, Jiang R, Yun J, Cao D. Why the abnormal phenomena of D-band center theory exist? A new BASED theory for surface catalysis and chemistry. *Chinese Journal of Catalysis*. 2024;64:44-53. doi:10.1016/S1872-2067(24)60100-2
